# PRISM: Prior-enhanced Inference for Spatial Transcriptomic Cell Type Mapping

**DOI:** 10.1093/bioinformatics/btag515

**Published:** 2026-07-23

**Authors:** Yiheng Xu, Xuehao Wang, Shuqi Liu, Congcong Ge, Xiang Chen, Yueming Wang, Bin Yu, Xiao-Ming Li

**Affiliations:** Department of Psychiatry of the Second Affiliated Hospital, Zhejiang University School of Medicine, Hangzhou, 310009, China; NHC and CAMS Key Laboratory of Medical Neurobiology, MOE Frontier Center of Brain Science and Brain-Machine Integration, School of Brain Science and Brain Medicine, Liangzhu Laboratory, Zhejiang University, Hangzhou, 310058, China; College of Computer Science and Technology, Zhejiang University, Hangzhou, 310027, China; College of Computer Science and Technology, Zhejiang University, Hangzhou, 310027, China; Department of Psychiatry of the Second Affiliated Hospital, Zhejiang University School of Medicine, Hangzhou, 310009, China; Nanhu Brain-Computer Interface Institute, Hangzhou, 311100, China; NHC and CAMS Key Laboratory of Medical Neurobiology, MOE Frontier Center of Brain Science and Brain-Machine Integration, School of Brain Science and Brain Medicine, Liangzhu Laboratory, Zhejiang University, Hangzhou, 310058, China; School of Software Technology, Zhejiang University, Hangzhou, 310027, China; College of Computer Science and Technology, Zhejiang University, Hangzhou, 310027, China; Nanhu Brain-Computer Interface Institute, Hangzhou, 311100, China; College of Computer Science and Technology, Zhejiang University, Hangzhou, 310027, China; Institute of Brain and Cognitive Science, School of Medicine, Hangzhou City University, Hangzhou, 310015, China; Department of Psychiatry of the Second Affiliated Hospital, Zhejiang University School of Medicine, Hangzhou, 310009, China; Nanhu Brain-Computer Interface Institute, Hangzhou, 311100, China; NHC and CAMS Key Laboratory of Medical Neurobiology, MOE Frontier Center of Brain Science and Brain-Machine Integration, School of Brain Science and Brain Medicine, Liangzhu Laboratory, Zhejiang University, Hangzhou, 310058, China

## Abstract

**Motivation:**

Cell type annotation in spatial transcriptomics (ST) is fundamental for deciphering complex tissue organization and spatially resolved biological processes. Most existing methods perform ST cell type annotation by transferring labels from single-cell RNA-seq (scRNA) data to ST data, but typically rely on weakly constrained representations that neglect structured spatial dependencies and treat marker gene selection as an isolated preprocessing step. This renders them vulnerable to substantial domain gaps as well as platform-specific noise, resulting in unstable predictions and limited biological interpretability.

**Results:**

To address these issues, we propose Prior-enhanced Inference for Spatial Transcriptomic Cell Type Mapping (PRISM), a novel three-stage framework integrating biological prior construction, pseudo-label generation, and multi-level ST refinement. First, PRISM constructs a cross-domain biological prior to explicitly extract marker genes to enforce positive biological discriminability. Next, it adopts a prior-enhanced self-training strategy, where scRNA-trained ensembles generate reliable pseudo-label candidates for ST data, serving as a robust anchor for cross-domain adaptation. Finally, the framework consolidates high-quality ensemble predictions selected via metric-guided evaluation, encodes spatial information, and optimizes the model under dual-directional biological constraints. Extensive experiments on eleven ST datasets across six platforms, two species, and multiple tissue contexts validate PRISM. Specifically, on the five labeled benchmarks, PRISM shows strong overall performance under both Accuracy and Macro-F1 evaluation across brain and non-brain tissues. Moreover, under fully label-free settings, PRISM achieves the best overall composite rank across all datasets, demonstrating strong robustness to domain shift and platform heterogeneity.

**Availability and implementation:**

PRISM is available at https://github.com/lilab-ai4s/PRISM and https://doi.org/10.5281/zenodo.20529683.

## 1 Introduction

Spatial transcriptomics (ST) (Cho *et al.* 2021) technologies enable measurement of gene expression within intact tissue structures, providing spatially resolved views of cellular organization and biological processes. A wide range of computational methods for spatial transcriptomics has accordingly emerged, including SpaMask (Min *et al.* 2025) for spatial-domain representation learning, mclSTExp (Min *et al.* 2024) for cross-modal expression prediction, and scstGCN (Xue *et al.* 2024) for single-cell-resolution inference from spot-based ST. By using these methods, ST has become a critical tool for probing tissue development, disease progression, and functional organization at the spatial level (Daly *et al.* 2025, Qian *et al.* 2025, Wang *et al.* 2025). However, transcriptomic signals captured by current ST platforms remain incomplete due to limited sequencing depth and restricted gene coverage, which is further exacerbated in cell-level settings and makes reliable cell type annotation difficult on ST data (Li *et al.* 2022, You *et al.* 2024). To address this challenge, two complementary strategies have emerged. The first is spatial integration, which aligns and integrates spatial omics data across slices, platforms, or modalities to enrich downstream analyses, with recent advances such as SpaCross (Fang and Min 2025) and SpaBatch (Niu *et al.* 2025). The second is reference-guided cell-type inference, which uses annotated scRNA-seq data to infer cellular identities or compositions in ST data, with representative methods such as Spatial-ID (Shen *et al.* 2022) and recent advances such as SpaDAMA (Huang *et al.* 2025), leveraging the high-quality annotated references available in scRNA-seq across diverse studies (Daly *et al.* 2025, Qian *et al.* 2025, Wang *et al.* 2025). The present work follows this reference-guided strategy, focusing specifically on per-cell categorical annotation at single-cell resolution. scRNA-seq technologies, including Smart-seq2 (Picelli *et al.* 2014), Drop-seq (Macosko *et al.* 2015), and 10x Genomics Chromium (Zheng *et al.* 2017), profile gene expression at single-cell resolution to generate such references. The target ST data spans diverse measurement platforms with distinct technical characteristics: *in situ* capture-based methods such as Visium (Ståhl *et al.* 2016), Slide-seqV2 (Stickels *et al.* 2021), Stereo-seq (Chen *et al.* 2022), and Slide-tags (Russell *et al.* 2024) sequence transcripts after spatial capture and provide broad transcriptome-wide or near-transcriptome-wide gene coverage, but typically at the cost of per-location cellular resolution, which can be partially recovered through cell segmentation; imaging-based methods such as MERFISH (Zhang *et al.* 2023), seqFISH+ (Eng *et al.* 2019), STARmap (Wang *et al.* 2018), and Xenium (Janesick *et al.* 2023) all rely on predefined gene panels and differ primarily in their decoding chemistry, while providing single-cell or near-single-cell resolution within a limited gene set. Although annotated scRNA-seq data provides a valuable reference foundation, the platform-specific heterogeneity and sparsity across diverse ST technologies still pose substantial challenges for cross-domain knowledge transfer.

Within this scRNA-to-ST transfer paradigm, existing approaches differ in their primary analytical objectives. Tangram (Biancalani *et al.* 2021) is an optimization-based method that aligns reference cells or cell-type-level profiles to spatial locations, depending on its operating mode. Spatial-ID (Shen *et al.* 2022) and DSCT (Xu *et al.* 2025) use deep-learning architectures for cell-type assignment. In contrast, RCTD (Cable *et al.* 2022), SpatialDWLS (Dong and Yuan 2021), and Cell2location (Kleshchevnikov *et al.* 2022) are primarily designed for cell-type composition estimation or deconvolution, producing continuous abundance or proportion estimates at each spatial location. For categorical comparison in this study, the dominant inferred cell type is used as the assigned label for these deconvolution methods. More specifically, RCTD estimates cell-type composition through a probabilistic deconvolution framework; SpatialDWLS first identifies candidate cell types at each spatial location and then applies dampened weighted least squares to estimate their relative proportions; Cell2location uses a Bayesian framework to infer spatial variation in cell-type abundance. Tangram minimizes reconstruction error to align reference and spatial data, whereas Spatial-ID and DSCT use neural-network-based models with graph and attention modules for cell-type assignment.

Beyond model architecture, gene selection is critical for defining the feature space in high-dimensional gene expression data, as it directly affects representation quality and downstream mapping performance. Most existing methods rely on traditional differential expression analysis (Love *et al.* 2014). For example, Tangram (Biancalani *et al.* 2021) uses t-tests, RCTD (Cable *et al.* 2022) applies log-fold change, and SpatialDWLS (Dong and Yuan 2021) uses enrichment analysis to identify marker genes. Beyond differential expression-based approaches, methods such as Cell2location (Kleshchevnikov *et al.* 2022) and Spatial-ID (Shen *et al.* 2022) incorporate manually curated marker genes. However, such strategies may not generalize well across tissues or platforms. Additionally, DSCT (Xu *et al.* 2025) identifies marker genes more effectively from a deep learning perspective, but their attention-based strategies introduce randomness and tend to be sensitive to hyperparameters. Despite these variations, all the aforementioned methods typically treat marker gene selection as an isolated preprocessing step, leaving biological knowledge implicit and static.

In summary, despite substantial progress in both mapping models and gene selection strategies, existing methods still commonly suffer from one or more of the following challenges (Shen *et al.* 2022, Xu *et al.* 2025): (i) most methods rely on weakly constrained representations that fail to capture cell identity; (ii) existing models often neglect structured spatial dependencies, limiting the effective use of spatial context; and (iii) marker gene selection is typically treated as an isolated preprocessing step, leaving biological knowledge implicit and disconnected from the inference process, which limits cross-platform generalization. These limitations emphasize the urgent need for a more efficient, scalable, and biologically interpretable framework for cell type mapping on ST data.

To tackle these challenges, we propose Prior-enhanced Inference for Spatial Transcriptomic Cell Type Mapping (PRISM), a biology-aware framework designed for robust, cross-domain cell type mapping on ST data. Specifically, PRISM is a three-stage pipeline comprising cross-domain biological prior construction, metric-guided pseudo-label adaptation, and multi-level ST refinement. First, PRISM establishes cross-domain biological priors to enforce positive biological discriminability within input representations. Second, it trains an ensemble of prior-enhanced inference networks to generate multiple sets of pseudo-labels, which act as distributional anchors to stabilize scRNA-to-ST adaptation under domain shift. Finally, PRISM refines these pseudo-labels through metric-guided selection and aggregation to train a model, integrating spatial inductive biases with dual-directional biological constraints to promote local homogeneity and suppress residual spurious correlations. Experiments on eleven ST datasets across multiple platforms and species demonstrate that PRISM yields accurate, stable, and anatomically faithful annotations.

## 2 Materials and methods

In this section, we introduce the components of the proposed PRISM framework and its overall pipeline.

**Notations.** Let $N_{\text{sc}}$ and $N_{\text{st}}$ denote the numbers of cells in scRNA and ST matrix, while $G_{\text{sc}}$ and $G_{\text{st}}$ are the numbers of genes in each. The raw data of scRNA and ST are formulated as two matrices $X_{\text{sc}}^{\text{raw}}\in R^{N_{\text{sc}}\times G_{\text{sc}}}$ and $X_{\text{st}}^{\text{raw}}\in R^{N_{\text{st}}\times G_{\text{st}}}$, respectively, and each element of matrices is the raw count of each gene in the corresponding cell. Given *C* cell types, each scRNA cell is associated with a cell type label, forming a labeled dataset $D_{\text{sc}}^{\text{raw}}=(X_{\text{sc}}^{\text{raw}},Y_{\text{sc}})$, where $Y_{\text{sc}}\in {\left\{1,\ldots ,C\right\}}^{N_{\text{sc}}}$. Different from scRNA data, each ST cell *i* is unlabeled and paired with a spatial coordinate vector ${\text{loc}}_{i}=(u_{i},v_{i})$, forming the spatial dataset $D_{\text{st}}^{\text{raw}}=(X_{\text{st}}^{\text{raw}},{\text{Loc}}_{\text{st}})$, where ${\text{Loc}}_{\text{st}}\in R^{N_{\text{st}}\times 2}$.

### 2.1 Overview of PRISM

**Figure 1:**
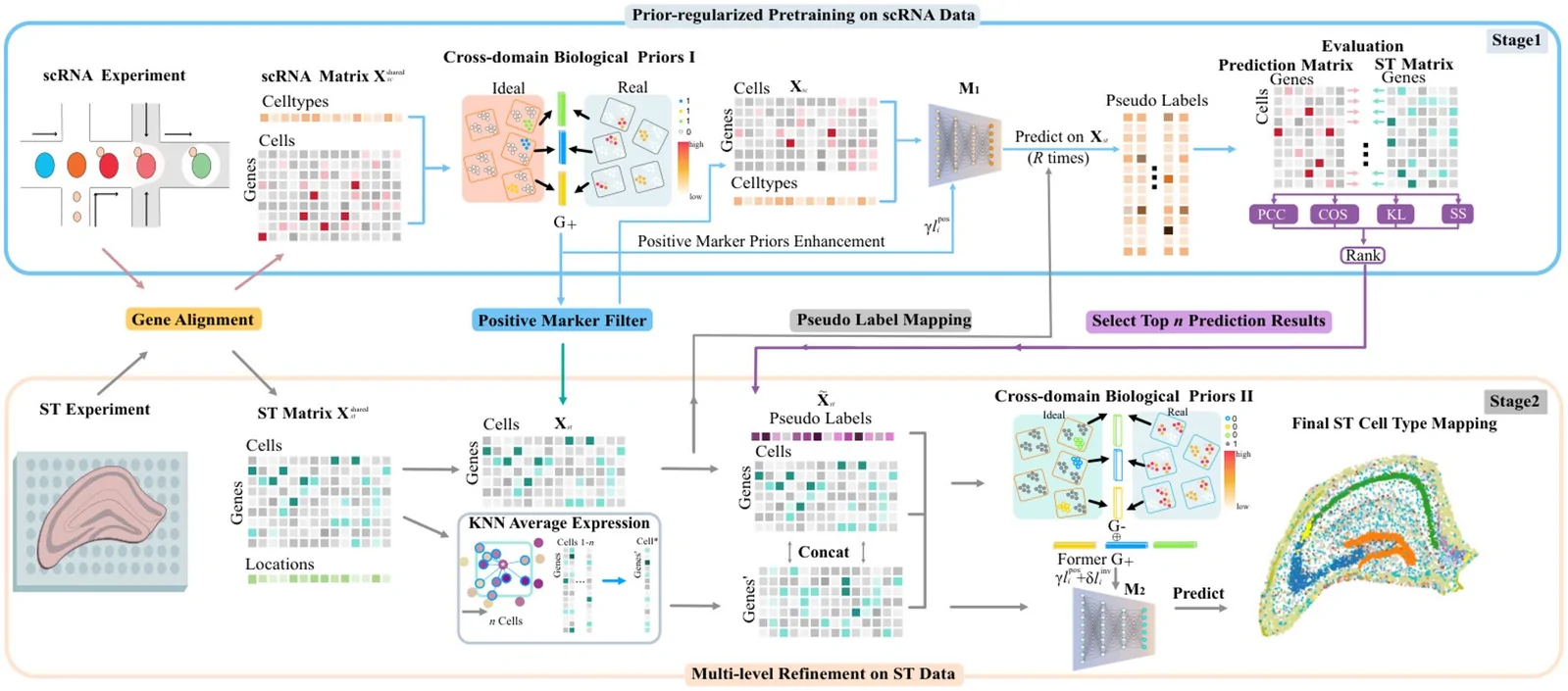
The overview of the Prior-enhanced Inference for Spatial Transcriptomic Cell Type Mapping (PRISM) framework.

Figure 1 schematically illustrates the workflow of PRISM, which combines cross-domain biological prior construction as a preprocessing step with two training stages: prior-enhanced pseudo-label generation (Stage 1) and multi-level refinement (Stage 2).

**Gene alignment and cross-domain biological prior construction (preprocessing).** We first align scRNA and ST data to a shared gene set $G_{\text{shared}}$ and apply library-size normalization to establish a comparable feature space across modalities. We then construct cross-domain biological priors by selecting cell-type-specific positive marker genes within this shared space, yielding harmonized marker-restricted feature matrices $X_{\text{sc}}$ and $X_{\text{st}}$. These markers are further integrated into the inference procedure as class-specific logit adjustments.

**Stage 1: Prior-enhanced pseudo-label generation.** We train an ensemble of inference models, $M_{1}$, with positive marker prior enhancement as defined in Equation (6), on the labeled source data $X_{\text{sc}}$ using diverse initializations, where each model in the ensemble is trained with a different random seed controlling network weight initialization and stochastic aspects of training, while the architecture, marker-gene set, and hyperparameters are held fixed. The resulting models are subsequently applied to $X_{\text{st}}$ to produce *R* pseudo-label sets, yielding reliable pseudo-label candidates that are robust to domain shift. Here, a pseudo-label denotes a cell-type label predicted for an intrinsically unlabeled ST cell by the scRNA-trained model ensemble; following the semi-supervised learning convention, these predicted labels are subsequently reused as supervision targets for the refinement model $M_{2}$.

**Stage 2: Multi-Level ST refinement.** First, the *R* pseudo-label sets are evaluated using a multi-dimensional metric framework, and the top-*n* ranked sets are aggregated to construct a high-quality ST dataset ${\hat{D}}_{\text{st}}$ for supervising the refinement model. Next, to spatially augment the feature representation, each cell is concatenated with the mean expression of its *k*-nearest neighbors, forming the spatially augmented input ${\tilde{X}}_{\text{st}}\in R^{N_{\text{total}}\times 2G}$ for the refined model $M_{2}$. Finally, to suppress residual noise and prevent misclassification, $M_{2}$ is trained with both positive marker gene injection and inverse marker gene noise compensation to yield spatially coherent cell type mapping.

### 2.2 Gene alignment and cross-domain biological prior construction

To bridge the domain gap between scRNA and ST data, we establish biologically grounded priors to guide cross-domain cell type mapping. Unlike conventional methods that treat marker gene selection as an isolated preprocessing step, we formulate type–gene relevance priors that serve as explicit biological constraints for the downstream inference framework. Specifically, to align the input modalities, both $X_{\text{sc}}^{\text{raw}}$ and $X_{\text{st}}^{\text{raw}}$ for scRNA and ST data require a systematic two-step feature construction process.

**Gene alignment as preprocessing.** To align the feature space of scRNA and ST data, we construct a shared gene space. Specifically, genes are aligned by taking the intersection of the two gene sets $X_{\text{sc}}^{\text{raw}}$ and $X_{\text{st}}^{\text{raw}}$, thus the shared gene set $G_{\text{shared}}$ is formulated as


(1) $$G_{\text{shared}}=G_{\text{sc}}\cap G_{\text{st}},$$

where $G_{\text{sc}}$ and $G_{\text{st}}$ denote the gene set of scRNA and ST data, respectively. By selecting the gene expression of the genes in the $G_{\text{shared}}$, the raw data of scRNA and ST are aligned and share a consistent input space. Then, the aligned data of scRNA and ST are transformed to $X_{\text{sc}}^{\text{shared}}\in R^{N_{\text{sc}}\times G_{\text{shared}}}$ and $X_{\text{st}}^{\text{shared}}\in R^{N_{\text{st}}\times G_{\text{shared}}}$ by library size normalization and natural logarithm transformation, as detailed in Section 7, Normalization, available as supplementary data at *Bioinformatics* online.

**Construction of cell-type-specific marker priors.** Biological prior construction begins after gene alignment, with cell-type-specific marker information incorporated into the inference and refinement procedure. Within the shared gene space, we aim to formalize intrinsic gene-cell-type relationships into quantifiable biological priors, specifically targeting positive marker genes that define cellular identity (Zhang *et al.* 2019), and encode gene–cell-type associations as explicit constraints for downstream inference. Specifically, for each cell type *c*, the ideal binary gene expression vector $e_{c}\in {\left\{0,1\right\}}^{N_{\text{sc}}}$ is formulated as


(2) $${\left(e_{c}\right)}_{i}=\{\begin{array}{cc} 1 & \text{if}\;Y_{\text{sc}}^{i}=c,\\ 0 & \text{otherwise}, \end{array}$$

where $Y_{\text{sc}}^{i}$ denotes the type of cell *i*. Let $g_{g}={\left[X_{\text{sc}}^{\text{shared}}(1,g),\ldots ,X_{\;\text{sc}}^{\text{shared}}(N_{\text{sc}},g)\right]}^{⊤}$ be the vector of gene expression for gene *g* in all cells, where $X_{\text{sc}}^{\text{shared}}(i,g)$ denotes the gene expression of gene *g* in cell *i*. The correlation between the gene *g* and the cell type *c* is measured by cosine similarity between $e_{c}$ and $g_{g}$ following COSG (Dai *et al.* 2022), which is formulated as


(3) $$s_{c,g}=\text{COS}\;\left(e_{c},g_{g}\right)\in [0,1].$$

$s_{c,g}$ approaching +1 indicates the gene expression is positively correlated with cell type *c*, which means the gene expression of *g* is crucial to cell type *c*. Thus, the positive marker gene set $M_{+,c}$ for cell type *c* is defined as the set of genes with the top-*m* similarity scores in $S_{c}={\left[s_{c,g}\right]}_{g=1}^{G_{\text{shared}}}$. We define the overall positive marker set as $M_{+}=\overset{C}{\underset{c=1}{\cup }}M_{+,c}$. Since marker selection is performed within the shared gene space, $M_{+}\subseteq G_{\text{shared}}$ by construction. The final feature set is therefore defined as $G=M_{+}$, yielding *G* genes. Finally, the gene expression of each cell in $X_{\text{sc}}^{\text{shared}}$ and $X_{\text{st}}^{\text{shared}}$ is filtered by positive marker gene set to form the input data $X_{\text{sc}}$ and $X_{\text{st}}$, which are used for subsequent training and inference. Note that positive marker genes are selected from the annotated scRNA-seq reference rather than from the target ST labels, and the resulting marker features are then transferred to independent ST datasets for inference and evaluation. The labeled ST cells are therefore never used to select these features, so the evaluation assesses transfer to spatial cells that are unseen during marker selection.

### 2.3 Prior-enhanced inference network

To explicitly incorporate the biological priors constructed in the previous stage, we use a four-layer fully connected network with a residual skip connection from the input to the output logits as the inference model. Biologically, we integrate positive marker gene information at the logit level, thereby regularizing the training process and facilitating effective knowledge transfer across modalities.

**Architecture of the inference network.** The architecture described in this paragraph refers to the scRNA-trained inference model $M_{1}$. The inference network preserves the original gene-expression signal through a residual skip path while learning non-linear feature transformations in its hidden layers. Given the gene expression vector $z_{i}$ of cell *i* from the scRNA or ST data, a network with four fully connected layers and a residual skip connection is trained to map $z_{i}$ to the cell type *c*. The final fully connected projection $W_{\text{fc}}$ maps the last hidden representation $h_{i}^{\left(4\right)}$ to the cell-type logit space, producing the fully connected branch logits $l_{i}^{\text{fc}}$. The forward process is formulated as


$$\begin{array}{ccc} & h_{i}^{\left(1\right)}=\sigma \left(W_{1}z_{i}+b_{1}\right), & h_{i}^{\left(2\right)}=\sigma \left(W_{2}h_{i}^{\left(1\right)}+b_{2}\right),\\ & h_{i}^{\left(3\right)}=\sigma \left(W_{3}h_{i}^{\left(2\right)}+b_{3}\right), & h_{i}^{\left(4\right)}=\sigma \left(W_{4}h_{i}^{\left(3\right)}+b_{4}\right),\\ & l_{i}^{\text{fc}}=W_{\text{fc}}h_{i}^{\left(4\right)}, & l_{i}^{\text{skip}}=W_{\text{skip}}z_{i}, \end{array}$$

where ${\left\{W_{m},b_{m}\right\}}_{m=1}^{4}$, $W_{\text{skip}}$ and $W_{\text{fc}}$ are the parameters of the model and σ(·) is the ReLU activation function. The logits used for cell type mapping are


(4) $$l_{i}=l_{i}^{\text{fc}}+l_{i}^{\text{skip}}.$$

This residual formulation establishes a direct path to raw gene expression vectors via $l_{i}^{\text{skip}}$, thereby preserving critical marker gene signals and allowing the deep layers to focus exclusively on learning the non-linear refinements required for precise classification (He *et al.* 2016). The final logits are passed through a softmax to produce class probabilities, and the model is trained with the categorical cross-entropy loss.

**Enforcement of biological priors.** To explicitly incorporate marker gene-level biological knowledge into the inference process, we introduce a mechanism that leverages confirmed cell-type signatures to regularize the decision boundary. Formally, we enhance the guidance of cell-type-specific marker genes on cell type mapping by integrating their corresponding gene expression


(5) $$l_{i}^{\text{pos}}=z_{i}G_{+},\;\text{where}\;{\left(G_{+}\right)}_{c,g}=\{\begin{array}{cc} 1 & \text{if}\;g\in M_{+,c},\\ 0 & \text{otherwise}. \end{array}$$

Thus the logits is formulated as


(6) $$l_{i}=l_{i}^{\text{fc}}+l_{i}^{\text{skip}}+\gamma l_{i}^{\text{pos}},$$

where γ>0 is a hyperparameter controlling the weight of marker gene guidance. By imposing these enhancements directly at the logit level, the model preserves explicit biological evidence instead of absorbing it implicitly into hidden representations, ensuring that predictions remain biologically consistent.

### 2.4 Multi-level ST refinement

The inference network in Stage 1 treats cells as independent entities and does not explicitly model spatial correlations, while the scRNA-to-ST domain gap can introduce background noise that affects direct inference on ST data. To address these issues, we use a multi-level refinement strategy with three coordinated steps: *refinement of pseudo-labels* via ensemble-based selection, *refinement of spatial features* through local context aggregation, and *refinement of the inference model* via inverse marker gene noise compensation.

**Refinement of pseudo-labels: Ensemble-based selection strategy.** Due to domain shifts, scRNA-trained models often yield fluctuating predictions when directly applied to ST data. To mitigate this uncertainty and obtain robust supervision, we adopt an ensemble-based selection strategy. Specifically, the inference network $M_{1}$ is trained with diverse initializations to obtain *R* candidate pseudo-label sets ${\left\{Y_{st,r}\right\}}_{r=1}^{R}$. Subsequently, to identify the most reliable candidate from these sets, we establish a multi-dimensional metric evaluation that assesses the consistency between ST and scRNA data as a robust proxy for the quality of pseudo-labels.

We score each set against the reference using four similarity metrics, namely Pearson correlation coefficient (PCC), cosine similarity (COS), Kullback–Leibler divergence (KL), and structural similarity (SS), motivated by the premise that accurately mapped cells should exhibit transcriptomic profiles consistent with their reference counterparts (Cable *et al.* 2022, Li *et al.* 2022). Detailed metric definitions and per-set score aggregation are provided in Section 8, Evaluation Metrics, available as supplementary data at *Bioinformatics* online. For each candidate *r* and metric M∈{PCC, COS ,KL,SS}, we obtain a per-metric rank ${\text{rank}}_{M}(r)$ (1 = best) and define the composite rank as:


(7) $$\text{Rank}(r)=\frac{1}{4}\sum_{M}{\text{rank}}_{M}(r),$$

which is subsequently used to select the top-*n* pseudo-label sets with the lowest ${\text{Rank}}_{r}$. Then the high-quality pseudo-label set is constructed by aggregating the samples from these selected rounds:


(8) $${\hat{D}}_{\text{st}}=({\hat{X}}_{\text{st}},{\hat{\text{Loc}}}_{\text{st}},{\hat{Y}}_{\text{st}})=\underset{r\in R^{⋆}}{⊎}D_{\text{st}}^{r},$$

where $D_{\text{st}}^{r}=\left(X_{\text{st}},\;{\text{Loc}}_{\text{st}},\;Y_{\text{st}\;,r}\right)$ and $R^{⋆}$ is the set of pseudo-label set indices with the lowest *n* ${\text{Rank}}_{r}$ values. Here, ⊎ denotes the multiset union of the selected pseudo-labeled datasets, so that each ST cell contributes one pseudo-labeled instance from every selected round, paired with the pseudo-label assigned in that round. The selected predictions are therefore kept as ensemble-derived supervision for $M_{2}$ rather than collapsed into a single consensus label by voting or averaging.

By consolidating predictions from the most consistent models, this ensemble aggregation effectively filters out stochastic noise inherent in single-round inference, yielding a robust training set with high-confidence labels on ST data.

**Refinement of spatial features: Local context aggregation.** With reliable pseudo-labels established, we proceed to refine the feature representation by incorporating spatial context. Moreover, to ensure computational efficiency, instead of using complex learnable graph structures, we introduce a deterministic, non-parametric spatial prior. Concretely, we augment each cell’s own expression with that of its local neighborhood, where proximal cells tend to share similar profiles (Svensson *et al.* 2018). As a result, this approach effectively captures microenvironmental consistency while preserving the distinctiveness of cell-type-specific marker genes.

Specifically, for each cell *i* in ST data, we concatenate its gene expression vector $z_{i}$ with the mean expression vector of its *k* nearest spatial neighbors, which is formulated as


(9) $${\bar{z}}_{N_{k}(i)}=\frac{1}{k}\sum_{j\in N_{k}(i)}z_{j},\;{\tilde{z}}_{i}=[z_{i}\;\|\;{\bar{z}}_{N_{k}(i)}],$$

where $N_{k}(i)$ denotes the set of its *k* nearest spatial neighbors, $z_{j}$ denotes gene expression vector of neighbor *j*, [·‖·] is the concatenation operator. Consequently, we construct the spatially augmented ST matrix ${\tilde{X}}_{\text{st}}\in R^{\left(n\times N_{\text{st}})\times (2\times G\right)}$ and the ST dataset $D_{\text{st}}=\left({\tilde{X}}_{\text{st}},\;{\hat{\text{Loc}}}_{\text{st}},\;{\hat{Y}}_{\text{st}}\right)$. This design introduces a deterministic local smoothness prior that is sufficient to capture spatial coherence, avoiding the optimization instability and scalability issues often associated with graph-based spatial cell type mapping models.

**Refinement of inference model: Inverse marker gene noise compensation.** To mitigate the non-specificity of positive marker genes shared among similar cell types and suppress residual spurious correlations after spatial refinement, we introduce an inverse marker gene noise compensation mechanism that penalizes predictions inconsistent with cell-type-specific negative signatures. To implement this, the refined model $M_{2}$ is trained on spatially augmented ST data by imposing additional negative constraints, thereby improving its ability to distinguish similar cell types under domain shift.

In this context, inverse marker genes correspond to genes that show consistently low similarity to, or minimal expression in, the target cell type, serving as negative constraints to prevent misclassification of transcriptomically similar cell types. Specifically, we identify the inverse marker gene set $M_{-,c}$, consisting of the genes that have the bottom-*m* similarity, in the cosine similarity matrix $S^{\text{st}}=[s_{c,g}^{\text{st}}]\in R^{C\times (2\times G)}$ on refined ST data. Thus, the inverse guide matrix $G_{-}$ is defined as


(10) $${\left(G_{-}\right)}_{c,g}=\{\begin{array}{cc} 1 & \text{if}\;g\in M_{-,c},\\ 0 & \text{otherwise}. \end{array}$$

For the prediction on the ST data, the final logits are formulated as


(11) $$l_{i}=l_{i}^{\text{fc}}+l_{i}^{\text{skip}}+\gamma l_{i}^{\text{pos}}+\delta l_{i}^{\text{inv}},$$

where $l_{i}^{\text{inv}}=-{\tilde{z}}_{i}G_{-}$, and δ>0 controls the impact of the inverse marker gene noise compensation. This term explicitly and consistently penalizes candidate assignments that conflict with cell-type-specific negative signatures, thereby mitigating spurious signals introduced during spatial cell type mapping.

## 3 Results

We evaluated PRISM on eleven publicly available ST datasets covering six platforms (MERFISH, Slide-seqV2, STARmap, Stereo-seq, Xenium *In Situ*, and CosMx) and two species. Five datasets carry ground-truth cell-type annotations and are used for label-based benchmarks: MERFISH mouse hippocampus (HIP), olfactory bulb (OB), and cortex (CTX) from the Allen Brain Cell Atlas; MERFISH mouse liver paired with Tabula Muris Senis; and CosMx human hepatocellular carcinoma (HCC) paired with matched single-cell annotations from the SPATCH resource (Ren *et al.* 2025). The remaining six datasets are evaluated under the label-free protocol: Stereo-seq mouse cerebellum (CB) and HIP, Slide-seqV2 mouse HIP, STARmap mouse HIP, MERFISH human CTX, and Xenium human breast cancer (BC). A detailed description of each dataset, including data sources and matched scRNA-seq references, is provided in Section 10, Datasets, available as supplementary data at *Bioinformatics* online. All datasets are processed with a uniform pipeline of library-size normalization and log1p transformation; preprocessing details are described in Section 7, Normalization, available as supplementary data at *Bioinformatics* online.

### 3.1 Evaluation metrics

**Label-based evaluation metric.** For ST datasets with ground truth, we report accuracy as the fraction of cells whose predicted labels match the ground truth provided in the original datasets. The provenance of these reference annotations, including how the labels for the labeled MERFISH brain benchmarks were derived from the Allen Brain Cell Atlas, is described in Section 4, Reference Label Provenance, available as supplementary data at *Bioinformatics* online.

**Label-free evaluation metric.** For ST datasets lacking ground truth, we use five label-free metrics covering error, rank correlation, concordance, and distribution distance: Root Mean Squared Error (RMSE), Spearman rank correlation (Spearman ρ), Kendall rank correlation (Kendall τ), Lin’s Concordance Correlation Coefficient (CCC), and Jensen–Shannon Divergence (JSD) (Jin and Liu 2021, Li *et al.* 2022, 2023, Yan and Sun 2023, Tao *et al.* 2024, Xu *et al.* 2025). For each dataset, methods are ranked under each metric (1=best), and the composite rank is the arithmetic mean of the five per-metric ranks. Detailed definitions are provided in Section 8, Evaluation Metrics, available as supplementary data at *Bioinformatics* online.

### 3.2 Quantitative results for cell type mapping

**Performance on label-based benchmarks.** As shown in Table 1, on the five labeled benchmarks spanning brain (HIP, OB, CTX_mouse_) and non-brain tissues (MERFISH Liver, CosMx HCC) across two ST platforms, PRISM attains the highest Accuracy on four of the five datasets (0.906, 0.893, 0.818, and 0.799 on HIP, CTX_mouse_, Liver, and HCC, respectively) and the highest Macro-F1 on three of the five (0.535, 0.641, and 0.759 on HIP, OB, and CTX_mouse_). The combined Accuracy and Macro-F1 evaluation indicates that PRISM remains competitive across both brain and non-brain tissues and across heterogeneous ST platforms. The stability of PRISM across repeated runs is further examined in the ablation analysis in Section 2, Ablation Study, available as supplementary data at *Bioinformatics* online.

**Table 1:** Accuracy and Macro-F1 comparison of competing methods on five label-based datasets.^a^

Dataset	**PRISM**		**DSCT**		**SpatialDWLS**		**Tangram**		**Cell2location**		**Spatial-ID**		**RCTD**	
	Acc	MF1	Acc	MF1	Acc	MF1	Acc	MF1	Acc	MF1	Acc	MF1	Acc	MF1
MERFISH HIP	0.906	0.535	0.850	0.361	0.189	0.151	0.752	0.295	0.287	0.090	0.327	0.042	0.872	0.502
MERFISH OB	0.928	0.641	0.838	0.459	0.791	0.217	0.610	0.297	0.682	0.170	0.160	0.046	0.952	0.615
MERFISH CTX _mouse_	0.893	0.759	0.794	0.643	0.559	0.514	0.834	0.656	0.880	0.751	0.762	0.463	0.840	0.669
MERFISH Liver	0.818	0.535	0.806	0.477	0.811	0.366	0.662	0.443	0.638	0.595	0.141	0.008	0.741	0.459
CosMx HCC	0.799	0.221	0.709	0.150	0.791	0.273	0.121	0.085	0.672	0.261	0.710	0.111	0.496	0.052

a MF1 denotes Macro-F1; best per metric in **bold**.

**Robustness in label-free and cross-platform settings.** In label-free settings, PRISM ranks among the *top-performing* methods across evaluation metrics and achieves the best overall composite rank. As shown in Table 2, across eleven datasets spanning six spatial platforms, two species, and diverse tissue types ranging from brain regions to breast cancer and hepatocellular carcinoma, PRISM exhibits stable performance across heterogeneous ST tissues, indicating robustness to variations in tissue organization and sequencing platforms. Across all eleven datasets, PRISM achieves the best composite rank in each dataset (per-metric values supporting these ranks are provided in Table S8, available as supplementary data at *Bioinformatics* online). Because the composite rank is computed within each dataset before averaging across metrics, this summary reduces the influence of dataset-specific scale differences among RMSE, rank correlations, concordance, and distributional-distance scores. Collectively, these findings establish PRISM as a robust framework for label-free spatial cell type mapping that generalizes effectively across heterogeneous platforms.

**Table 2:** Composite rank of competing methods on all datasets.^a^

**Datasets**			**Methods**						
Platform	Tissue	Species	PRISM	Tangram	RCTD	DSCT	Cell2location	Spatial-ID	SpatialDWLS
MERFISH	HIP	Mouse	**1.2**	2.4	2.4	4.2	4.8	6.0	7.0
MERFISH	OB	Mouse	**1.2**	3.4	1.8	4.6	5.8	5.4	5.8
MERFISH	CTX	Mouse	**1.2**	1.8	5.0	3.6	3.4	6.0	7.0
MERFISH	CTX	Human	**1.6**	3.2	3.6	2.2	6.0	6.0	5.4
Stereo-seq	HIP	Mouse	**2.2**	2.3	4.7	3.2	3.3	5.9	6.4
STARmap	HIP	Mouse	**1.8**	3.0	5.2	3.0	5.8	2.8	6.4
Slide-seq	HIP	Mouse	**1.8**	2.8	6.4	5.2	3.6	4.0	4.2
Stereo-seq	CB	Mouse	**1.7**	1.9	3.4	4.0	7.0	4.4	5.6
MERFISH	Liver	Mouse	**1.0**	6.0	4.4	2.0	6.2	5.4	3.0
Xenium	BC	Human	**1.2**	2.8	7.0	4.8	2.6	3.6	6.0
CosMx	HCC	Human	**1.8**	3.6	4.0	2.8	5.4	6.4	4.0

a The best result in each dataset is in **bold**.

**Figure 2:**
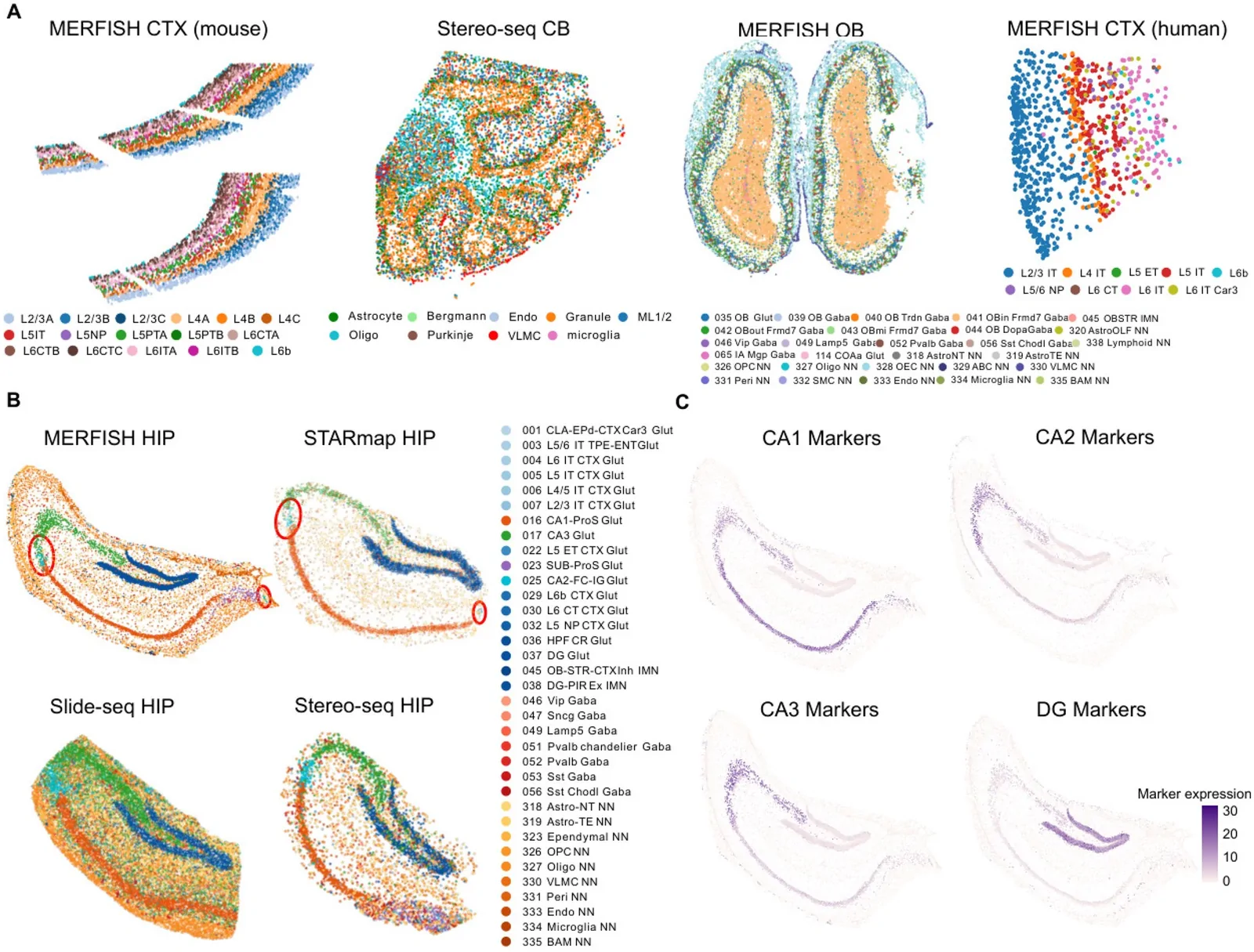
Biological validity and spatial coherence of PRISM predictions. (A) Spatial distribution of cell types in the CB, OB, CTX_mouse_, and CTX_human_ predicted by PRISM. (B) Spatial distribution of cell types in the HIP from different platforms predicted by PRISM (outlined regions indicate spatial similarity between CA2 and FC). (C) Marker gene distribution across HIP subregions.

**Biological validity and insights revealed by PRISM.** PRISM consistently recapitulates canonical spatial organization across multiple brain regions, providing strong evidence of biological validity. In the cortex, predicted cell types exhibit a clear six-layer (L1-L6) laminar structure, faithfully reproducing known cytoarchitecture. In the CB, PRISM cleanly delineates the M1/2 zones, while in the OB, it resolves the characteristic layered architecture, explicitly distinguishing the olfactory nerve, glomerular, external plexiform, mitral cell, internal plexiform, and granule layers (Fig. 2A). Furthermore, visualization of the combined expression of top marker genes for cornu ammonis subregions (CA1-3) and dentate gyrus (DG) shows precise spatial correspondence with anatomical landmarks (Fig. 2C), confirming accurate region-specific mapping. Notably, PRISM also reveals a clear spatial resemblance between CA2 and the fasciola cinerea (FC) populations in HIP, consistent with prior transcriptomic studies (Laeremans *et al.* 2013) reporting their strong molecular conservation (Fig. 2B). These laminar and subregional patterns are recovered consistently across MERFISH, Stereo-seq, STARmap, and Slide-seqV2 hippocampal slices (Fig. 2B), suggesting that the recovered organization reflects intrinsic biology rather than platform-specific artefacts. Together, these observations indicate that PRISM’s predictions not only achieve quantitative accuracy but also align with anatomical landmarks across cortical, hippocampal, cerebellar, and olfactory systems.

Beyond recovering known anatomical patterns, PRISM also reduces noise under domain shift. For example, on the MERFISH HIP dataset, PRISM assigns fewer off-target cortical subtypes (i.e. CTX-related labels mistakenly placed on HIP cells) relative to competing methods. PRISM yields the fewest spurious assignments (46 cells), followed by RCTD (75 cells), whereas Cell2location and SpatialDWLS each produce more than 1800 such assignments (Fig. S1A, available as supplementary data at *Bioinformatics* online). This reduction highlights an advantage of PRISM in resolving cross-platform hallucination, where models force-fit ambiguous signals to dominant source labels. Mechanistically, such resilience is attributable to the dual-directional biological constraints: by combining positive marker gene enforcement with inverse marker gene penalties, PRISM not only confirms cell identity but also rejects contradictory predictions, suppressing false-positive transfers that purely positive-learning models may fail to detect.

**Downstream cell-cell communication analysis.** To demonstrate that PRISM’s annotations support downstream interpretation beyond cell-type identification, we used the PRISM-derived cell-type labels on the HIP MERFISH dataset as input to LARIS (Dai *et al.* 2025), a single-cell-resolution spatial ligand-receptor interaction tool. LARIS identified 68 spatially specific ligand-receptor pairs and 2355 significant sender-receiver cell-type interactions (FDR < 0.05). Among the top-ranked interactions, the *Pdyn–Oprk1* (prodynorphin-κ-opioid receptor) pair recovers the known hippocampal dynorphin signaling axis. The sender-receiver analysis (Fig. S1B, available as supplementary data at *Bioinformatics* online) shows the strongest signals sent from dentate gyrus glutamatergic neurons (DG Glut) to DG Glut and CA3 glutamatergic neurons (CA3 Glut), consistent with reported dynorphin-mediated modulation of mossy fiber–CA3 transmission (Weisskopf *et al.* 1993). Consistently, the spatial distribution of the per-cell interaction score (Fig. S1C, available as supplementary data at *Bioinformatics* online) is confined to the dentate gyrus and the adjacent CA3 subfield. These results illustrate that PRISM cell-type maps can serve as a starting point for biologically meaningful downstream analyses, rather than as classification outputs alone.

## 4 Discussion

PRISM has two important limitations that we summarize here together with the corresponding future directions. First, PRISM operates in a closed-set reference-guided setting, in which the label space is restricted to the cell types represented in the scRNA-seq reference. Cell types or states absent from the reference cannot currently be identified as novel classes. PRISM exposes the per-cell softmax output as uncalibrated per-class assignment probabilities, which should not be interpreted as calibrated confidence estimates. Developing calibrated uncertainty estimation together with an explicit open-set rejection mechanism for novel cell types is therefore an important direction for future work. Second, PRISM is designed for ST data at or near single-cell resolution. For spot-based platforms such as 10x Visium, the softmax output of PRISM could in principle provide per-class assignment scores for each spatial location, but these scores should not be interpreted as native spot-level deconvolution proportions because each spot may contain mixtures of multiple cell types. In such settings, dedicated deconvolution methods are usually a more natural choice. Extending the framework to produce native spot-level compositional outputs is therefore a natural future direction that would broaden its applicability to lower-resolution platforms. These extensions would require replacing the single-label supervision with objectives that model uncertainty or cell-type mixtures, while retaining the marker-prior and spatial-refinement components that make PRISM effective at single-cell resolution.

## Supplementary Material

btag515_Supplementary_Data

## Data Availability

No new primary sequencing data were generated in this study. All datasets analysed in this study are publicly available from the sources described and cited in Supplementary Section S10. The PRISM source code, benchmarking scripts, and supporting materials are available at https://github.com/lilab-ai4s/PRISM and are archived at Zenodo at https://doi.org/10.5281/zenodo.20529683.
