# Supplementary material for: PRISM: Prior-enhanced Inference for Spatial Transcriptomic Cell Type Mapping
Source: Bioinformatics. 2026 Jul 23;42(8):btag515. doi: 10.1093/bioinformatics/btag515 (PMC13430658; doi:10.1093/bioinformatics/btag515)
Supplement: btag515_Supplementary_Data [file btag515_supplementary_data.pdf]

Supplementary Material

S1. Results Visualization of All Methods

To provide a more qualitative comparison, we present additional figures showing the performance of all evaluated methods across the benchmark datasets. The following figures supplement the quantitative results in the main text and facilitate a more comprehensive understanding of method behaviors (Figure S2).

S2. Ablation Study

We summarize the five PRISM architectural components evaluated in this study. **Skip** denotes the skip connection in the inference network. **Pseudo-label Refinement (PR)** performs ensemble-based selection of high-confidence pseudo-labels from multiple initializations using the four similarity criteria described in Section S8. **Spatial Priors (SP)** corresponds to the local context aggregation module, in which each cell's expression is augmented with that of its k nearest neighbors. **Positive Marker Prior (POS)** adds a logit boost based on positive marker genes for each cell type. **Inverse Marker Prior (Inv)** adds a logit penalty based on inverse marker genes. Each configuration in Table S1 is named according to the components enabled on top of the MLP backbone.

To directly evaluate the contribution of each component, we report two complementary analyses on the three labeled benchmarks (HIP, $\mathrm{CTX_{mouse}}$, and OB). The first analysis (rows 1–6 of Table S1) examines the standalone effects of Skip, POS, and SP added individually to the MLP backbone, and quantifies the contribution of PR in its intended operating context by comparing PR+POS+Skip with POS+Skip. The second analysis (rows 6–9 of Table S1) uses a 2×2 factorial design to evaluate the individual and joint effects of SP and Inv on top of the PR+POS+Skip configuration.

Component-wise analysis of Skip, POS, SP, and PR (rows 1–6 of Table S1). Starting from the pure MLP baseline, both Skip and POS provide clear standalone improvements across all three datasets. Compared with the Baseline, Skip increases Accuracy by +27.7, +9.0, and +19.8 percentage points on HIP, $\mathrm{CTX_{mouse}}$, and OB, respectively, while POS increases Accuracy by +24.7, +7.9, and +16.5 percentage points. We additionally introduced a POS+Skip configuration (row 5) to quantify the contribution of PR in its intended operating context. Adding PR on top of POS+Skip (row 6 vs row 5) further increases Accuracy by +1.7, +4.6, and +4.4 percentage points and Macro-F1 by +3.4, +6.7, and +6.9 percentage points on HIP, $\mathrm{CTX_{mouse}}$, and OB, respectively. In contrast, SP applied alone does not improve over the Baseline, indicating that neighborhood context alone is insufficient to improve performance in these benchmarks.

SP×Inv 2×2 factorial analysis (rows 6–9 of Table S1). On top of the PR+POS+Skip configuration, adding Inv alone produces small and mixed changes relative to row 6: Accuracy changes by +0.5, −0.7, and −0.2 percentage points, while Macro-F1 changes by −0.9, −0.5, and +1.5 percentage points on HIP, $\mathrm{CTX_{mouse}}$, and OB, respectively. Adding SP without Inv decreases performance, particularly in Macro-F1, with changes of −8.1, −5.5, and −12.2 percentage points across the three datasets. In contrast, enabling SP and Inv together yields the best Accuracy and Macro-F1 on all three datasets, improving over PR+POS+Skip by +1.8, +4.6, and +0.5 percentage points in Accuracy and by +1.4, +2.2, and +7.2 percentage points in Macro-F1. These results suggest that SP and Inv have complementary effects in the refined model. This pattern is consistent with their intended roles: SP introduces neighborhood context that may also incorporate mixed local signals, whereas Inv is designed to suppress signals inconsistent with the assigned cell type.

Overall, Skip and POS show clear standalone benefits, PR provides additional improvement when applied to the POS+Skip pathway for which it is designed, and SP and Inv contribute most effectively when used jointly in the refined model. Among all evaluated configurations, full PRISM achieves the highest Accuracy and Macro-F1 on HIP, $\mathrm{CTX_{mouse}}$, and OB, reaching Accuracy values of 0.906, 0.893, and 0.928 and Macro-F1 values of 0.535, 0.759, and 0.641, respectively.

S3. Inverse Marker Contribution

Comparing full PRISM with the inverse-marker-removed variant (row 9 vs row 8 of Table S1) shows that removing Inv decreases Accuracy by 6.8, 5.0, and 7.3 percentage points and Macro-F1 by 9.5, 7.7, and 19.4 percentage points on HIP, $\mathrm{CTX_{mouse}}$, and OB, respectively. These drops indicate that the inverse-marker term provides a substantial contribution, particularly to class-balanced performance.

To further evaluate this effect at the cell-type level, Table S2 reports a HIP MERFISH per-cell-type comparison between Full PRISM and the inverse-marker-removed variant. Per-cell-type accuracy is computed as recall for each ground-truth cell type, and per-cell-type F1 is reported alongside it. The largest gains are observed in several inhibitory and non-neuronal populations, including RHP-COA Ndnf Gaba, Lamp5 Lhx6 Gaba, Peri NN, Microglia NN, Vip Gaba, and Sst Gaba, indicating that inverse markers help suppress misleading local signals for specific biologically meaningful cell types.

S4. Reference Label Provenance

For the labeled MERFISH benchmarks (HIP, OB, $\mathrm{CTX_{mouse}}$), the reference cell-type annotations are taken from dataset-provided annotations based on the Allen Institute mouse brain cell-type taxonomy released through the Allen Brain Cell Atlas (Yao et al., 2023). The Allen taxonomy is established from large-scale scRNA-seq and snRNA-seq atlases through unsupervised clustering followed by expert curation, and cell-type labels for individual MERFISH cells are assigned by mapping onto this curated transcriptomic taxonomy with anatomical information and quality-control filtering by the Allen Institute. Accuracy and Macro-F1 values reported in our benchmarks therefore reflect agreement with this curated reference taxonomy rather than absolute biological ground truth. Because the same reference annotations are used to evaluate all compared methods on each dataset, any residual uncertainty in the labels applies equally to all methods and does not favor PRISM specifically.

For the MERFISH mouse liver benchmark, the reference cell-type annotations are taken from the dataset-provided labels released together with the MERFISH liver atlas, in which cell-type identities were originally assigned by the authors through transcriptomic clustering of the MERFISH measurements followed by marker-guided assignment to canonical mouse liver cell types. We use these dataset-provided labels without modification, and they are applied identically

across all compared methods. The paired scRNA-seq reference is the mouse liver subset of the Tabula Muris Senis FACS atlas (The Tabula Muris Consortium, 2020), whose annotations were curated by the original consortium and are likewise used as released.

For the CosMx human hepatocellular carcinoma (HCC) benchmark, the reference cell-type annotations are taken from the matched single-cell annotations released through the SPATCH resource (Ren et al., 2025), in which CosMx cells were annotated by the original authors against a paired single-cell reference from the same study with expert curation of tumor and tumor-microenvironment cell types. As for the MERFISH benchmarks, these dataset-provided labels serve as the evaluation reference for all compared methods on this dataset.

Across all five labeled benchmarks, Accuracy and Macro-F1 therefore reflect agreement with the cell-type taxonomies provided by the original data releases rather than absolute biological ground truth, and the same reference labels are applied to every compared method.

S5. Feature-Set Substitution

To separate the contribution of the PRISM framework from the contribution of the PRISM marker set, we re-ran all six competing methods (RCTD, Tangram, Cell2location, Spatial-ID, SpatialDWLS, and DSCT) with the PRISM positive marker set as their input feature space, on the three labeled brain MERFISH benchmarks (HIP, OB, and $\mathrm{CTX}_{\mathrm{mouse}}$). The marker set is the same as in the main comparison: 414, 427, and 113 unique genes across 50, 30, and 8 cell types for HIP, OB, and $\mathrm{CTX}_{\mathrm{mouse}}$, respectively. Both the native-marker and PRISM-marker runs use the same ground-truth labels and the same evaluation pipeline, so the Δ values in Table S3 are directly comparable. For DSCT, whose marker selection is performed by an attention module trained jointly with the inference network, we used a four-layer fully connected backbone with a residual skip connection (the architecture that DSCT reduces to once its attention module is removed) and fed it the PRISM marker set as input, mirroring the protocol applied to the other five methods.

Two patterns emerge from Table S3. First, the PRISM marker set is itself a high-quality feature set: it yields substantial gains for several competing methods in specific method-dataset pairs, most notably Spatial-ID and SpatialDWLS on HIP and OB (up to +61.1 percentage points in Accuracy) and DSCT across the three datasets. Second, under matched-marker conditions, PRISM remains among the top-performing methods across the three labeled benchmarks. These results indicate that although the PRISM marker set improves several competing methods, marker selection alone does not fully account for PRISM's performance.

S6. Sensitivity Analysis

We analyze the sensitivity of PRISM to several key design choices, including spatial neighborhood size k, similarity-derived marker gene number m, pseudo-label aggregation rounds n, and marker gene prior weighting parameters γ, δ. Note that the sensitivity analyses below evaluate model-internal stability across hyperparameters and therefore reuse the four Stage-2 selection criteria (SS, KL, Cosine, Pearson); these criteria are not used as performance metrics in the label-free benchmark of Section 3.1, which is reported under an independent panel of five metrics (RMSE, Spearman ρ, Kendall τ, CCC, JSD).

Sensitivity to spatial neighborhood size. We examine the sensitivity of PRISM to the spatial neighborhood size used in the refinement stage. Using the MERFISH HIP and $\mathrm{CTX}_{\mathrm{mouse}}$ datasets, we vary the number of nearest neighbors $k \in \{0, 1, 5, 10, 15, 20\}$ while keeping all other components fixed. As shown in Tables S9 and S10, spatial refinement remains stable across neighborhood sizes, with best or near-best performance around $k = 10$–15. Performance improves noticeably from small neighborhoods and gradually saturates around $k = 10$–15, without degradation at larger scales. Notably, the overall variation across different k values ($k \geq 5$) remains limited, suggesting that the refinement process does not depend on a precisely tuned neighborhood parameter, with all evaluated measures remaining within narrow ranges. Together, these results indicate that PRISM effectively leverages spatial context while maintaining robustness across a spectrum of neighborhood scales.

Sensitivity to marker gene set size across platforms. We further find that although PRISM does not depend on a specific marker gene cardinality, different spatial transcriptomics platforms exhibit preferences for different marker gene set sizes. As shown in Tables S11–S15, the marker gene configuration achieving the strongest performance varies across technologies. Specifically, Stereo-seq and MERFISH favor moderate marker gene sets (e.g., 30 marker genes), whereas STARmap and Slide-seq achieve optimal results with larger marker gene sets (e.g., 45 marker genes). We believe this difference stems from the distinct measurement characteristics of each platform, which influence how informative signals are aggregated when constructing similarity-guided marker gene priors. Consequently, different technologies naturally benefit from different marker gene scales, even though PRISM remains stable across a broad range of marker gene configurations. Based on these results, we recommend selecting the marker gene set size in a platform-adaptive manner, guided by platform-specific characteristics. Notably, performance remains stable within a reasonable range around these values, indicating that PRISM supports flexible marker gene configurations without requiring fine-grained tuning.

Sensitivity to pseudo-label aggregation rounds. PRISM refines pseudo-labels by aggregating predictions from multiple independently initialized models. On the MERFISH hippocampus dataset, we vary the number of aggregation rounds n from 1 to 5. As shown in Table S16, using a single round yields noticeably inferior performance, indicating that pseudo-labels remain unstable under insufficient ensemble diversity. Increasing the number of rounds n from 1 to 3 leads to substantial improvement, after which performance largely saturates, with only marginal gains observed beyond 3 rounds. Further increasing aggregation depth offers diminishing returns while incurring additional computational costs. Overall, these results suggest that a moderate level of ensemble diversity is sufficient to stabilize pseudo-label quality, balancing accuracy with computational efficiency.

Sensitivity to marker gene prior weighting parameters. We further analyze the sensitivity of PRISM to the weighting parameters γ and δ, which control the contributions of positive marker gene-consistent and inverse marker gene-inconsistent signals, respectively. As shown in Table S17, varying γ and δ across a broad range results in consistently stable performance, with accuracy maintaining

a high level (around 0.90). Configurations that balance positive and inverse contributions (e.g., $\gamma = 5, \delta = 5$) achieve strong results (accuracy = 0.9063), while other nearby parameter settings yield comparable outcomes. This observation indicates that PRISM is robust to variations in prior weighting and does not rely on a complex tuned scheme to effectively leverage biological constraints.

Summary. Overall, the sensitivity analysis demonstrates that PRISM is robust to key design choices and hyperparameters. Spatial refinement exhibits stable behavior across a wide range of neighborhood sizes, while pseudo-label aggregation achieves stability with moderate ensemble diversity. Similarity-guided marker gene priors generalize reliably across platforms, where marker gene set size and weighting parameters admit flexible configurations without fine-grained tuning. These results confirm that the performance gains of PRISM arise from its principled integration of spatial inductive biases and biological priors, rather than from hyperparameter overfitting.

S7. Normalization

Library size normalization. For each cell i with raw count $d_{i,g}$ of gene g, the library size is $L_i = \sum_{g=1}^{G_{\text{shared}}} d_{i,g}$, and raw counts are rescaled to a fixed total of 10^4 transcripts per cell:

$$\tilde{d}_{i,g} \;=\; 10^4 \, \frac{d_{i,g}}{L_i}. \tag{12}$$

Log transformation. The library-normalized counts are then log-transformed with a pseudo-count of 1:

$$x_{i,g} \;=\; \log\!\big(1 + \tilde{d}_{i,g}\big), \tag{13}$$

where $\log(\cdot)$ denotes the natural logarithm. Then, the aligned data of scRNA and ST are transformed to $X_{\text{sc}}^{\text{shared}} \in \mathbb{R}^{N_{\text{sc}} \times G_{\text{shared}}}$ and $X_{\text{st}}^{\text{shared}} \in \mathbb{R}^{N_{\text{st}} \times G_{\text{shared}}}$.

S8. Evaluation Metrics

To quantitatively assess the consistency between predicted ST profiles and reference scRNA profiles, we employ four statistical similarity metrics: Pearson correlation coefficient (PCC), cosine similarity (COS), Kullback–Leibler divergence (KL), and structural similarity (SS). We denote the average gene expression $\mathbf{z}_{\text{sc}}^c$ of the cell type c in scRNA data as:

$$\mathbf{z}_{\text{sc}}^c = \frac{1}{n_c} \sum_{i \in I_c^{\text{sc}}} \mathbf{x}_i^{\text{sc}} \;\in \mathbb{R}^d, \tag{14}$$

where I_c^{sc} is the set of cells belonging to cell type c, and n_c is the number of single cells in I_c^{sc}. $x_i^{\text{sc}} \in \mathbb{R}^d$ denotes the d-genes, pre-processed expression vector of single cell i in the reference dataset. And we denote the gene expression $\mathbf{z}_{\text{st}}^i$ of the cell i with pseudo-label c in ST data. Then, the $\mathbf{z}_{\text{sc}}^c$ is compared with each $\mathbf{z}_{\text{st}}^i$, accumulating four metrics including Pearson Correlation Coefficient (**PCC**), Cosine Similarity (**COS**), Kullback–Leibler divergence (**KL**), Structural Similarity (**SS**):

$$\text{PCC}(\mathbf{z}_{\text{sc}}^c, \mathbf{z}_{\text{st}}^i) = \frac{\sum_j (\mathbf{z}_{\text{sc}}^{c,j} - \mu_{\text{sc}})(\mathbf{z}_{\text{st}}^{i,j} - \mu_{\text{st}})}{\sqrt{\sum_j (\mathbf{z}_{\text{sc}}^{c,j} - \mu_{\text{sc}})^2}\,\sqrt{\sum_j (\mathbf{z}_{\text{st}}^{i,j} - \mu_{\text{st}})^2}}; \tag{15}$$

$$\text{COS}(\mathbf{z}_{\text{sc}}^c, \mathbf{z}_{\text{st}}^i) = \frac{(\mathbf{z}_{\text{sc}}^c)^\top \mathbf{z}_{\text{st}}^i}{\|\mathbf{z}_{\text{sc}}^c\|_2 \, \|\mathbf{z}_{\text{st}}^i\|_2}; \tag{16}$$

$$\text{KL}\big(\mathbf{p} \parallel \mathbf{q}\big) = \sum_{j=1}^{d} p_j \log \frac{p_j}{q_j}, \quad \mathbf{p} = \frac{\mathbf{z}_{\text{sc}}^{c,j}}{\|\mathbf{z}_{\text{sc}}^{c,j}\|_1}, \; \mathbf{q} = \frac{\mathbf{z}_{\text{st}}^{i,j}}{\|\mathbf{z}_{\text{st}}^{i,j}\|_1}; \tag{17}$$

$$\text{SS}(\mathbf{z}_{\text{sc}}^c, \mathbf{z}_{\text{st}}^i) = \frac{(2\mu_{\text{sc}}\mu_{\text{st}} + C_1)(2\sigma_{\text{sc,st}} + C_2)}{(\mu_{\text{sc}}^2 + \mu_{\text{st}}^2 + C_1)(\sigma_{\text{sc}}^2 + \sigma_{\text{st}}^2 + C_2)}. \tag{18}$$

Here, j denotes the element of the two expression vectors. μ_{sc} and μ_{st} represent the respective means; $\sigma_{\text{sc,st}}$ is the cross-variance; σ_{sc}^2 and σ_{st}^2 denote the marginal variances. C_1 and C_2 are stability constants used in the SS formula. For KL divergence, the two expression vectors are first normalized by their ℓ_1 norms to convert them into probability distributions $\mathbf{p}$ and $\mathbf{q}$, respectively.

For pseudo-label set r, we aggregate these per-cell scores into a single score per metric. Specifically, we first compute a type-wise average over all ST cells assigned to cell type c, resulting in a score $\hat{s}_{c,M}^r$ for metric $M \in \{\text{PCC, COS, KL, SS}\}$:

$$\hat{s}_{c,M}^r \;=\; \frac{1}{|I_c|} \sum_{i \in I_c} M(\mathbf{z}_{\text{sc}}^c, \mathbf{z}_{\text{st}}^i), \tag{19}$$

where $I_c = \{i \mid 1 \le i \le N_{\text{st}},\ Y_{\text{st},r}^i = c\}$, $Y_{\text{st},r}^i$ denotes the pseudo-label of cell i in set r, and $|\cdot|$ denotes the size of the set. We then average these type-wise scores to obtain a global consistency score s_M^r for set r under metric M:

$$s_M^r \;=\; \frac{1}{C} \sum_{c=1}^{C} \hat{s}_{c,M}^r. \tag{20}$$

The global score s_M^r is the per-set, per-metric aggregate used in the main text to compute the per-metric rank $\text{rank}_M(r)$ and the composite rank $\text{Rank}(r)$.

The four metrics above (PCC, COS, KL, SS) are also used as ranking criteria inside PRISM's Stage 2 pseudo-label refinement. To make the label-free evaluation independent of the selection step, we introduce five additional metrics that do not reuse the exact criteria (PCC, COS, KL, and SS) employed for pseudo-label selection. The five metrics are Root Mean Squared Error (**RMSE**) (Yan and Sun, 2023; Li et al., 2023), Spearman rank correlation (**Spearman** ρ) (Li et al., 2022), Kendall rank correlation (**Kendall** τ) (Kendall, 1938), Lin's Concordance Correlation Coefficient (**CCC**) (Lin, 1989; Jin and Liu, 2021), and Jensen–Shannon Divergence (**JSD**) (Lin, 1991; Yan and Sun, 2023). They cover four different families of measures, namely absolute error, rank correlation, joint precision and accuracy, and symmetric distribution distance. Together, the five metrics form an evaluation panel that does not overlap with the criteria used for pseudo-label selection.

RMSE measures the absolute reconstruction error between the observed ST expression and the reference cell-type mean profile. Spearman ρ and Kendall τ measure rank agreement between the two expression vectors. Kendall τ is based on concordant and discordant gene pairs and is more conservative than Spearman ρ. CCC measures both correlation and bias from the 45° line, so it

penalizes systematic shifts in mean or scale. JSD is a symmetric and bounded distributional distance, which is different from the asymmetric and unbounded KL divergence used during selection. The five metrics are defined as follows.

$$\mathrm{RMSE}(\mathbf{z}_{\mathrm{sc}}^{c}, \mathbf{z}_{\mathrm{st}}^{i}) = \sqrt{\tfrac{1}{d}\sum_{j=1}^{d}\big(\mathbf{z}_{\mathrm{sc}}^{c,j} - \mathbf{z}_{\mathrm{st}}^{i,j}\big)^{2}}; \tag{21}$$

$$\rho(\mathbf{z}_{\mathrm{sc}}^{c}, \mathbf{z}_{\mathrm{st}}^{i}) = \mathrm{PCC}\big(\mathrm{rank}(\mathbf{z}_{\mathrm{sc}}^{c}),\ \mathrm{rank}(\mathbf{z}_{\mathrm{st}}^{i})\big); \tag{22}$$

$$\tau(\mathbf{z}_{\mathrm{sc}}^{c}, \mathbf{z}_{\mathrm{st}}^{i}) = \frac{n_{\mathrm{c}} - n_{\mathrm{d}}}{\binom{d}{2}}; \tag{23}$$

$$\mathrm{CCC}(\mathbf{z}_{\mathrm{sc}}^{c}, \mathbf{z}_{\mathrm{st}}^{i}) = \frac{2\,\sigma_{\mathrm{sc,st}}}{\sigma_{\mathrm{sc}}^{2} + \sigma_{\mathrm{st}}^{2} + (\mu_{\mathrm{sc}} - \mu_{\mathrm{st}})^{2}}; \tag{24}$$

$$\mathrm{JSD}(\mathbf{p} \,\|\, \mathbf{q}) = \tfrac{1}{2}\,\mathrm{KL}\big(\mathbf{p} \,\|\, \mathbf{m}\big) + \tfrac{1}{2}\,\mathrm{KL}\big(\mathbf{q} \,\|\, \mathbf{m}\big), \quad \mathbf{m} = \tfrac{1}{2}(\mathbf{p} + \mathbf{q}). \tag{25}$$

Here, n_{c} and n_{d} denote the number of concordant and discordant gene pairs between $\mathbf{z}_{\mathrm{sc}}^{c}$ and $\mathbf{z}_{\mathrm{st}}^{i}$. $\mathbf{p}$ and $\mathbf{q}$ are the ℓ_1-normalized distributions defined earlier. Lower values are better for RMSE and JSD. Higher values are better for Spearman ρ, Kendall τ, and CCC.

S9. Hyperparameters of Competing Methods

Competing Methods. SpatialDWLS: The method is scran, expression values are normalized, and the cluster column is leiden_clus; the Leiden clustering resolution is 0.4 and the number of iterations is 100. **RCTD:** The implementation is spacexr v2.0.0, the doublet mode is full, and the maximum number of reference cells is 10,000. **Tangram:** The mapping mode is cells, the density prior is RNA-count-based, and the number of training epochs is 100. **Cell2location:** The training runs for 300 epochs, the batch size is 300, the training size fraction is 1, and the learning rate is 0.002. **Spatial-ID:** The PCA dimension is 200, the graph k is 30, edge weighting is enabled, the number of training epochs is 200, and the losses are weighted with $w_{\mathrm{cls}} = 20$, $w_{\mathrm{dae}} = 1$, and $w_{\mathrm{gae}} = 1$. **DSCT:** For data filtering, the minimum counts are 0 for both scRNA and spatial data, the minimum cells per gene are 20; marker gene selection requires an expressed proportion of 0.1 and 30–60 genes per cell type are kept; model training runs for 200 epochs.

PRISM. We adopt a *dataset-specific* budget of class-specific marker genes, chosen according to the transcript depth of each platform: MERFISH-CTX$_{\mathrm{mouse}}$ 30, MERFISH-OB 30, MERFISH-HIP 30, Stereo-seq-HIP 30, Stereo-seq-CB 30, MERFISH-CTX$_{\mathrm{human}}$ 30, Xenium breast-cancer 30, MERFISH-Liver 30, CosMx-HCC 30, STARmap-HIP 45, and Slide-seqV2-HIP 45. This platform-adaptive configuration accounts for the distinct sequencing characteristics of each technology, with optimal values identified via a systematic grid search, which also serves as a sensitivity analysis to validate model robustness (see Supplementary Section S6, Sensitivity Analysis). All models are trained on 80% of the dataset, with the remaining 20% reserved for validation. In the first stage (M_1), we train 10 models with different random seeds; the best-performing runs are then carried forward to the second stage (M_2). Both stages run for 150 epochs and employ the Adagrad optimiser (learning rate 0.01) with hidden-layer widths {256, 128, 64, 32}. During optimization, marker gene and inverse marker gene logits are weighted by $\gamma = \delta = 5.0$. Spatial context is incorporated by concatenating each cell's expression with that of its $k = 15$ nearest neighbors.

S10. Datasets

ST datasets. We curate eleven publicly available ST datasets spanning **six** measurement platforms and two species, covering diverse gene-panel sizes and spatial-location counts. (1) *Stereo-seq* sagittal sections of the mouse **CB** and **HIP** are downloaded from the Brain Data Center[1]. (2) A mouse **HIP** *Slide-seqV2* dataset is obtained from the Broad Institute SCP948[2]. (3) A *STARmap* **HIP** slice[3]. (4) Four *MERFISH* datasets include **CTX$_{\mathbf{human}}$** and **CTX$_{\mathbf{mouse}}$** are from Dryad[4], as well as a mouse **HIP** slice and **OB** slice are released by the Allen Institute[5]. (5) A human **breast-cancer** *Xenium In Situ* dataset is available by 10x Genomics[6]. (6) A mouse **liver** *MERFISH* dataset is obtained from the figshare archive accompanying Liu *et al.* (*Life Science Alliance* 6(1):e202201701, 2022)[7]. (7) A human **hepatocellular carcinoma (HCC)** *CosMx* 6K dataset with matched single-cell annotations is obtained from the SPATCH resource (Ren et al., 2025)[8].

scRNA datasets. Matched scRNA references were obtained from the same repositories or companion studies: (1) **HIP** and (2) **OB** scRNA data are downloaded from the Allen Institute[5]; (3) **CB** scRNA data is acquired from the Brain Data Center[1]; (4) **CTX$_{\mathbf{human}}$** and **CTX$_{\mathbf{mouse}}$** scRNA data are obtained from Dryad[4]; (5) the **breast-cancer** scRNA data accompanying the *Xenium In Situ* dataset is released together with its paired ST library in the same 10x Genomics bundle[6]; (6) the **liver** scRNA reference is the mouse liver subset of the Tabula Muris Senis FACS atlas (The Tabula Muris Consortium, 2020); (7) the **HCC** scRNA reference is the matched single-cell dataset released together with its CosMx sample in the SPATCH resource[8].

S11. Compute Hardware

Hardware stack. All experiments are conducted on a Linux workstation equipped with an **AMD EPYC 7543** CPU (32 physical cores, 64 threads) and **two NVIDIA GeForce RTX 4090** GPUs, each providing 24 GB of GDDR6X memory. The system ran the `550.144.03` NVIDIA driver alongside `CUDA 12.4`. The PRISM script automatically detects CUDA and seamlessly falls back to CPU execution when no compatible GPU is available.

S12. Runtime and Computational Efficiency

As summarized in Table S18, PRISM achieves a balanced and resource-efficient inference profile on the MERFISH HIP dataset. It requires only **28.2 GB** RAM and **2.2 GB** GPU memory, while maintaining moderate CPU and GPU runtime, making it substantially lighter than memory-intensive probabilistic or graph-based alternatives. In contrast, several competing methods exhibit either excessive memory consumption or prohibitive runtime under

[1] https://www.braindatacenter.cn/datacenter/web/#/dataSet/details?id=1712760331765706754
[2] https://singlecell.broadinstitute.org/single_cell/study/SCP948
[3] https://zenodo.org/records/8041114
[4] https://datadryad.org/stash/dataset/doi:10.5061/dryad.x3ffbg7mw
[5] https://alleninstitute.github.io/abc_atlas_access/descriptions/MERFISH-C57BL6J-638850.html
[6] https://www.10xgenomics.com/products/xenium-in-situ/preview-dataset-human-breast
[7] https://figshare.com/projects/MERFISH_mouse_comparison_study/134213
[8] http://spatch.pku-genomics.org/

the same setting. Crucially, PRISM achieves this computational efficiency while simultaneously attaining the highest accuracy among all evaluated methods on this benchmark. These results indicate that PRISM offers an optimal balance between speed, resource usage, and predictive performance, enabling scalable spatial cell type mapping under realistic hardware constraints.

S13. Per-cell-type Performance

To complement the dataset-level Accuracy and Macro-F1 reported in the main text, we provide per-cell-type performance breakdowns on the three labeled MERFISH benchmarks. Per-cell-type F1 with class support is reported for HIP, OB, and $\mathrm{CTX}_{\mathrm{mouse}}$ in Tables S6, S7, and S5, respectively, allowing direct inspection of method behavior across both abundant and low-abundance cell types. To verify that PRISM remains effective across excitatory, inhibitory, and non-neuronal populations within the cortex, we additionally report a comprehensive CTX benchmark jointly evaluated on these three subsets in Table S4.

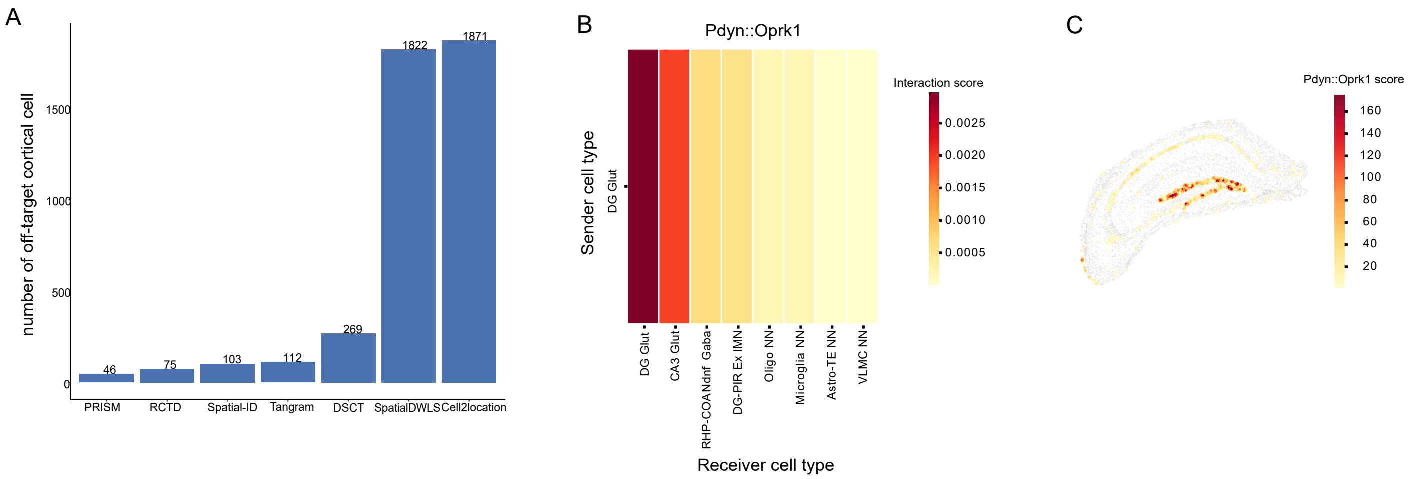

Figure S1 Qualitative validation of PRISM annotations on HIP MERFISH. (**A**) Number of off-target cortical cell mappings in HIP across competing methods; PRISM produces the fewest spurious cortical assignments. (**B**) Sender–receiver interaction heatmap for the top-ranked *Pdyn–Oprk1* (prodynorphin–κ-opioid receptor) pair from a cell-cell communication analysis on PRISM-derived cell types, with dentate gyrus glutamatergic neurons (DG Glut) as the dominant sender. (**C**) Spatial distribution of the per-cell LARIS *Pdyn–Oprk1* interaction score across the PRISM-annotated HIP section, enriched in the dentate gyrus and the adjacent CA3 subfield. Grey points denote all cells; coloured points denote cells with high interaction scores.

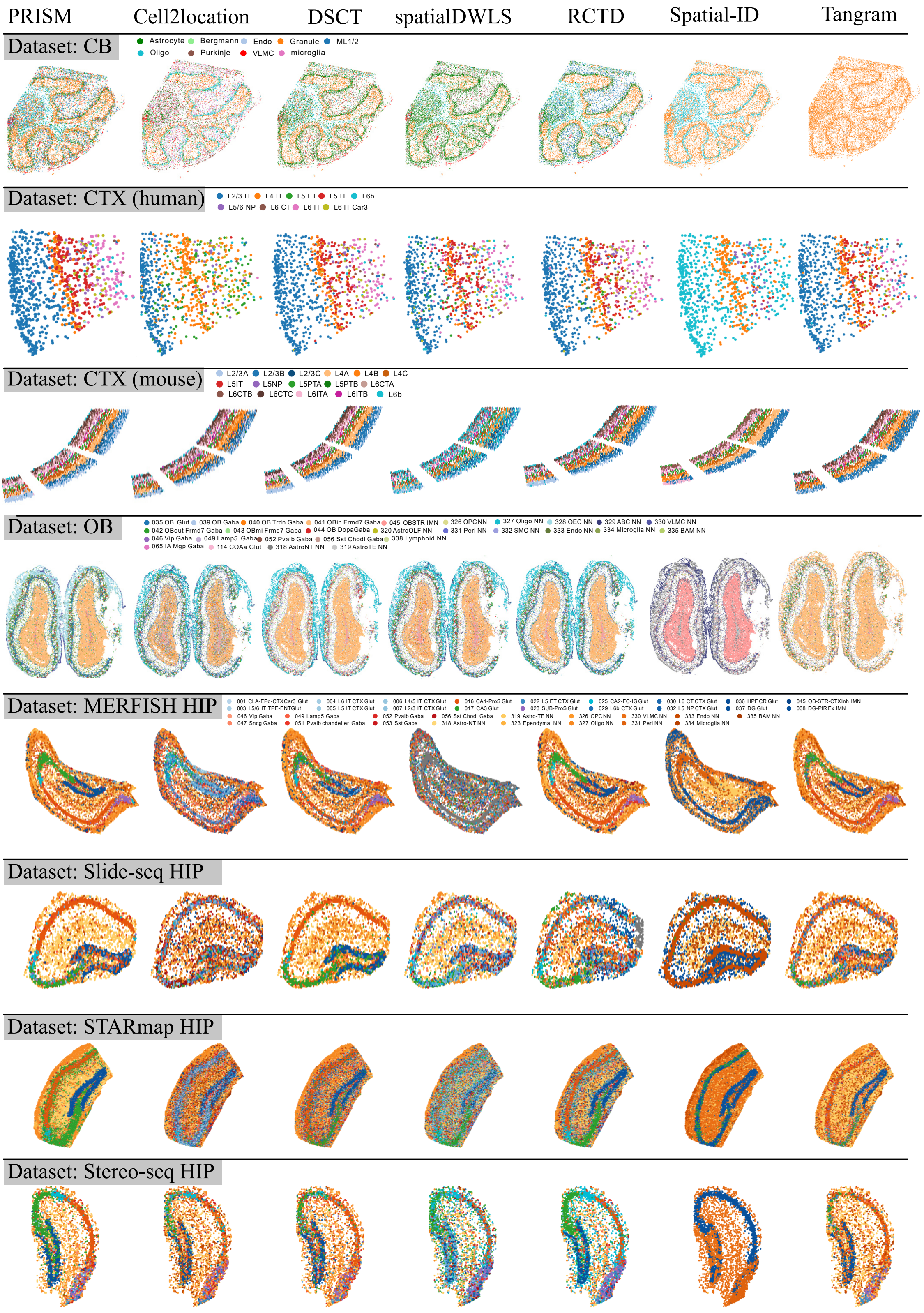

Figure S2 Performance of all methods on each dataset.

Table S1 Ablation study of PRISM components on label-based datasets (mean ± std over 10 runs).

#	Configuration	HIP (Acc / Macro-F1)	CTX$_{mouse}$ (Acc / Macro-F1)	OB (Acc / Macro-F1)
1	Baseline	$0.586_{\pm 0.131}$ / $0.167_{\pm 0.031}$	$0.696_{\pm 0.044}$ / $0.562_{\pm 0.026}$	$0.650_{\pm 0.020}$ / $0.193_{\pm 0.020}$
2	+Skip	$0.863_{\pm 0.005}$ / $0.430_{\pm 0.012}$	$0.786_{\pm 0.054}$ / $0.652_{\pm 0.043}$	$0.848_{\pm 0.031}$ / $0.431_{\pm 0.084}$
3	+POS	$0.833_{\pm 0.018}$ / $0.376_{\pm 0.013}$	$0.775_{\pm 0.030}$ / $0.631_{\pm 0.031}$	$0.815_{\pm 0.044}$ / $0.314_{\pm 0.069}$
4	+SP	$0.535_{\pm 0.078}$ / $0.154_{\pm 0.041}$	$0.692_{\pm 0.038}$ / $0.480_{\pm 0.072}$	$0.561_{\pm 0.031}$ / $0.133_{\pm 0.007}$
5	+POS+Skip	$0.871_{\pm 0.005}$ / $0.487_{\pm 0.013}$	$0.801_{\pm 0.024}$ / $0.670_{\pm 0.021}$	$0.879_{\pm 0.011}$ / $0.500_{\pm 0.031}$
6	+PR+POS+Skip	$0.888_{\pm 0.007}$ / $0.521_{\pm 0.008}$	$0.847_{\pm 0.009}$ / $0.737_{\pm 0.006}$	$0.923_{\pm 0.005}$ / $0.569_{\pm 0.015}$
7	+PR+POS+Skip+Inv	$0.893_{\pm 0.001}$ / $0.512_{\pm 0.013}$	$0.840_{\pm 0.010}$ / $0.732_{\pm 0.007}$	$0.921_{\pm 0.006}$ / $0.584_{\pm 0.014}$
8	+PR+POS+Skip+SP	$0.838_{\pm 0.003}$ / $0.440_{\pm 0.013}$	$0.843_{\pm 0.007}$ / $0.682_{\pm 0.008}$	$0.855_{\pm 0.001}$ / $0.447_{\pm 0.007}$
9	**+PR+POS+Skip+SP+Inv (Full PRISM)**	$\mathbf{0.906}_{\pm 0.004}$ / $\mathbf{0.535}_{\pm 0.010}$	$\mathbf{0.893}_{\pm 0.003}$ / $\mathbf{0.759}_{\pm 0.007}$	$\mathbf{0.928}_{\pm 0.003}$ / $\mathbf{0.641}_{\pm 0.016}$

Table S2 HIP MERFISH per-cell-type comparison between Full PRISM and the inverse-marker-removed variant. Only cell types with at least one correctly predicted cell under either configuration are shown.

Cell type	Support	Full Acc.	w/o Inv Acc.	△ Acc.	Full F1	w/o Inv F1	△ F1
001 CLA-EPd-CTX Car3 Glut	1	1.000	0.000	+1.000	1.000	0.000	+1.000
016 CA1-ProS Glut	881	0.965	0.991	-0.026	0.978	0.977	+0.001
017 CA3 Glut	601	0.977	0.967	+0.010	0.958	0.956	+0.001
023 SUB-ProS Glut	66	0.924	0.758	+0.167	0.938	0.855	+0.084
025 CA2-FC-IG Glut	63	0.762	0.905	-0.143	0.850	0.857	-0.008
029 L6b CTX Glut	5	0.600	0.400	+0.200	0.750	0.571	+0.179
030 L6 CT CTX Glut	3	0.333	0.333	0.000	0.400	0.500	-0.100
033 NP SUB Glut	55	0.927	0.800	+0.127	0.936	0.863	+0.073
036 HPF CR Glut	38	0.789	0.368	+0.421	0.882	0.538	+0.344
037 DG Glut	1333	0.977	0.975	+0.002	0.985	0.979	+0.006
038 DG-PIR Ex IMN	76	0.592	0.474	+0.118	0.732	0.643	+0.089
045 OB-STR-CTX Inh IMN	74	0.811	0.851	-0.041	0.784	0.663	+0.121
046 Vip Gaba	28	0.821	0.429	+0.393	0.793	0.585	+0.208
047 Sncg Gaba	29	0.621	0.586	+0.034	0.750	0.694	+0.056
048 RHP-COA Ndnf Gaba	51	0.882	0.039	+0.843	0.857	0.075	+0.782
049 Lamp5 Gaba	14	0.643	0.500	+0.143	0.750	0.467	+0.283
050 Lamp5 Lhx6 Gaba	56	0.768	0.071	+0.696	0.860	0.133	+0.727
051 Pvalb chandelier Gaba	4	1.000	0.250	+0.750	1.000	0.400	+0.600
052 Pvalb Gaba	58	0.897	0.483	+0.414	0.698	0.622	+0.076
053 Sst Gaba	84	0.786	0.583	+0.202	0.857	0.690	+0.167
056 Sst Chodl Gaba	3	1.000	1.000	0.000	1.000	1.000	0.000
213 SCsg Gabrr2 Gaba	13	0.846	0.462	+0.385	0.846	0.632	+0.215
318 Astro-NT NN	179	0.665	0.849	-0.184	0.723	0.764	-0.040
319 Astro-TE NN	1751	0.900	0.873	+0.027	0.882	0.739	+0.143
321 Astroependymal NN	12	0.250	0.250	0.000	0.353	0.375	-0.022
323 Ependymal NN	226	0.991	0.973	+0.018	0.968	0.948	+0.019
325 CHOR NN	6	0.500	0.333	+0.167	0.667	0.500	+0.167
326 OPC NN	157	0.981	0.713	+0.268	0.778	0.792	-0.014
327 Oligo NN	1623	0.948	0.902	+0.046	0.954	0.904	+0.050
329 ABC NN	6	0.833	0.000	+0.833	0.833	0.000	+0.833
330 VLMC NN	223	0.888	0.870	+0.018	0.818	0.795	+0.023
331 Peri NN	88	0.864	0.364	+0.500	0.844	0.525	+0.320
332 SMC NN	77	0.883	0.558	+0.325	0.840	0.711	+0.129
333 Endo NN	541	0.909	0.671	+0.238	0.899	0.767	+0.132
334 Microglia NN	159	0.692	0.340	+0.352	0.753	0.502	+0.251
335 BAM NN	8	0.875	0.625	+0.250	0.424	0.417	+0.008

Table S3 Feature-set substitution analysis. Accuracy and Macro-F1 of the six competing methods under their native marker selection (*Native*) and under the PRISM marker set (+*PRISM marker set*) on the three labeled brain MERFISH benchmarks.

Dataset	Method	Native (Acc / MF1)	+PRISM marker set (Acc / MF1)	Δ (Acc / MF1)
HIP	RCTD	0.872 / 0.502	0.891 / 0.499	+1.9 / −0.3
	Tangram	0.752 / 0.295	0.778 / 0.285	+2.6 / −1.0
	Spatial-ID	0.327 / 0.042	0.749 / 0.169	**+42.2** / **+12.7**
	Cell2location	0.287 / 0.090	0.265 / 0.085	−2.2 / −0.5
	SpatialDWLS	0.189 / 0.151	0.800 / 0.348	**+61.1** / **+19.7**
	DSCT	0.850 / 0.361	0.869 / 0.448	+1.9 / +8.7
	**PRISM**	—	**0.906** / **0.535**	—
OB	RCTD	0.952 / 0.615	0.957 / 0.608	+0.5 / −0.7
	Tangram	0.610 / 0.297	0.610 / 0.226	0.0 / −7.1
	Spatial-ID	0.160 / 0.046	0.729 / 0.226	**+56.9** / **+18.0**
	Cell2location	0.682 / 0.170	0.677 / 0.176	−0.5 / +0.6
	SpatialDWLS	0.791 / 0.217	0.799 / 0.255	+0.8 / +3.8
	DSCT	0.838 / 0.459	0.883 / 0.480	+4.5 / +2.1
	**PRISM**	—	**0.928** / **0.641**	—
CTX_{mouse}	RCTD	0.840 / 0.669	0.518 / 0.471	−32.2 / −19.8
	Tangram	0.834 / 0.656	0.862 / 0.762	+2.8 / +10.6
	Spatial-ID	0.762 / 0.463	0.771 / 0.713	+0.9 / **+25.0**
	Cell2location	0.880 / 0.751	0.871 / 0.824	−0.9 / +7.3
	SpatialDWLS	0.559 / 0.514	0.576 / 0.478	+1.7 / −3.6
	DSCT	0.794 / 0.643	0.810 / 0.759	+1.6 / +11.6
	**PRISM**	—	**0.893** / **0.759**	—

Table S4 Comprehensive CTX cell-type benchmark on the excitatory (L, 8 classes), inhibitory (I, 4 classes), and non-neuronal (O, 6 classes) subsets, evaluated jointly on 12,372 ST cells.

Method	L Acc	I Acc	O Acc	Combined Acc	MF1	Weighted-F1
PRISM	0.893	0.949	0.847	0.882	0.855	0.881
Cell2location	0.891	0.924	0.827	0.873	0.850	0.874
DSCT	0.850	0.946	0.821	0.864	0.840	0.862
Tangram	0.890	0.949	0.643	0.813	0.769	0.801
RCTD	0.852	0.900	0.420	0.713	0.756	0.775
SpatialDWLS	0.567	0.946	0.719	0.651	0.713	0.714
Spatial-ID	0.753	0.864	0.333	0.624	0.468	0.569

Table S5 Per-cell-type F1 on the comprehensive CTX benchmark across excitatory (L), inhibitory (I), and non-neuronal (O) subsets, evaluated jointly on 12,372 ST cells. Support is the number of ST cells per cell type.

Subset	Cell type	Support	PRISM	Cell2location	DSCT	Tangram	RCTD	SpatialDWLS	Spatial-ID
L	eL2/3.IT	2193	0.922	0.911	0.894	0.919	0.904	0.768	0.826
L	eL4/5.IT	1872	0.897	0.890	0.862	0.888	0.879	0.635	0.848
L	eL6.CT	1145	0.942	0.935	0.903	0.927	0.887	0.627	0.844
L	eL6.IT	682	0.827	0.842	0.764	0.863	0.868	0.729	0.590
L	eL5.ET	472	0.931	0.944	0.857	0.957	0.962	0.810	0.807
L	eL5.IT	468	0.693	0.758	0.619	0.723	0.750	0.517	0.074
L	eL6b	210	0.703	0.736	0.603	0.598	0.673	0.418	0.000
L	eNP	142	0.936	0.899	0.905	0.870	0.906	0.669	0.000
I	Pvalb	456	0.975	0.969	0.970	0.966	0.940	0.960	0.964
I	Sst	301	0.954	0.911	0.949	0.936	0.934	0.933	0.886
I	Vip	170	0.909	0.872	0.910	0.940	0.844	0.934	0.489
I	Lamp5	162	0.911	0.883	0.911	0.939	0.920	0.944	0.806
O	OD	1111	0.919	0.855	0.908	0.761	0.637	0.700	0.000
O	Astro	989	0.851	0.899	0.810	0.718	0.630	0.786	0.000
O	Endo	942	0.889	0.869	0.886	0.473	0.568	0.858	0.780
O	Micro	526	0.790	0.734	0.776	0.519	0.239	0.504	0.263
O	OPC	275	0.781	0.743	0.743	0.640	0.719	0.746	0.244
O	VLMC	256	0.563	0.653	0.496	0.213	0.339	0.288	0.000

Table S6 Per-cell-type F1 on the HIP MERFISH dataset across seven competing methods. Support is the number of ST cells per cell type.

Cell type	Support	PRISM	DSCT	Cell2location	Tangram	RCTD	SpatialDWLS	Spatial-ID
319 Astro-TE NN	1751	0.882	0.816	0.213	0.703	0.806	0.020	0.539
327 Oligo NN	1623	0.956	0.903	0.439	0.852	0.945	0.081	0.170
037 DG Glut	1333	0.988	0.928	0.237	0.978	0.991	0.514	0.053
016 CA1-ProS Glut	881	0.978	0.906	0.093	0.933	0.982	0.738	0.000
017 CA3 Glut	601	0.973	0.838	0.523	0.646	0.924	0.574	0.000
333 Endo NN	541	0.877	0.737	0.148	0.805	0.922	0.042	0.347
323 Ependymal NN	226	0.948	0.887	0.722	0.000	0.953	0.288	0.000
330 VLMC NN	223	0.813	0.712	0.408	0.326	0.855	0.149	0.163
318 Astro-NT NN	179	0.619	0.113	0.126	0.000	0.370	0.033	0.000
334 Microglia NN	159	0.871	0.716	0.362	0.580	0.824	0.000	0.008
326 OPC NN	157	0.845	0.741	0.308	0.617	0.798	0.057	0.000
151 TH Prkcd Grin2c Glut	90	0.000	0.000	0.000	0.000	0.000	0.000	0.000
331 Peri NN	88	0.764	0.633	0.017	0.651	0.889	0.000	0.097
053 Sst Gaba	84	0.852	0.717	0.343	0.810	0.880	0.460	0.000
332 SMC NN	77	0.841	0.527	0.227	0.513	0.859	0.136	0.012
038 DG-PIR Ex IMN	76	0.787	0.543	0.289	0.063	0.792	0.051	0.001
045 OB-STR-CTX Inh IMN	74	0.740	0.455	0.179	0.000	0.596	0.167	0.000
023 SUB-ProS Glut	66	0.926	0.531	0.138	0.873	0.961	0.287	0.000
025 CA2-FC-IG Glut	63	0.879	0.547	0.255	0.000	0.646	0.184	0.000
052 Pvalb Gaba	58	0.793	0.601	0.092	0.637	0.713	0.577	0.000
050 Lamp5 Lhx6 Gaba	56	0.881	0.803	0.162	0.899	0.938	0.635	0.000
033 NP SUB Glut	55	0.853	0.657	0.105	0.813	0.923	0.600	0.000
048 RHP-COA Ndnf Gaba	51	0.831	0.622	0.148	0.747	0.877	0.219	0.000
036 HPF CR Glut	38	0.884	0.753	0.339	0.410	0.898	0.000	0.000
047 Sncg Gaba	29	0.755	0.649	0.083	0.680	0.849	0.492	0.000
046 Vip Gaba	28	0.811	0.718	0.004	0.702	0.863	0.511	0.000
163 APN C1ql2 Glut	16	0.000	0.000	0.000	0.000	0.000	0.000	0.000
049 Lamp5 Gaba	14	0.823	0.620	0.051	0.842	0.923	0.696	0.000
213 SCsg Gabrr2 Gaba	13	0.723	0.428	0.285	0.000	0.444	0.556	0.000
321 Astroependymal NN	12	0.166	0.128	0.083	0.000	0.032	0.000	0.000
061 STR D1 Gaba	11	0.000	0.000	0.000	0.000	0.000	0.000	0.000
210 PRT Mecom Gaba	10	0.000	0.000	0.000	0.000	0.000	0.000	0.000
335 BAM NN	8	0.389	0.171	0.017	0.505	0.500	0.000	0.000
062 STR D2 Gaba	7	0.000	0.000	0.000	0.000	0.000	0.000	0.000
325 CHOR NN	6	0.089	0.432	0.134	0.000	0.857	0.250	0.000
329 ABC NN	6	0.755	0.327	0.179	0.000	0.800	0.000	0.000
029 L6b CTX Glut	5	0.679	0.454	0.054	0.000	0.889	0.150	0.000
202 PRT Tcf7l2 Gaba	5	0.000	0.000	0.000	0.000	0.000	0.000	0.000
203 LGv-SPFp-SPFm Nkx2-2 Tcf7l2 Gaba	5	0.000	0.000	0.000	0.000	0.000	0.000	0.000
051 Pvalb chandelier Gaba	4	0.537	0.265	0.011	0.432	0.444	0.000	0.000
030 L6 CT CTX Glut	3	0.140	0.207	0.009	0.000	0.500	0.667	0.000
056 Sst Chodl Gaba	3	1.000	0.690	0.108	0.050	0.857	0.000	0.000
032 L5 NP CTX Glut	2	0.000	0.011	0.000	0.000	0.000	0.000	0.000
164 APN C1ql4 Glut	2	0.000	0.000	0.000	0.000	0.000	0.000	0.000
168 SPA-SPFm-SPFp-POL-PIL-PoT Sp9 Glut	2	0.000	0.000	0.000	0.000	0.000	0.000	0.000
001 CLA-EPd-CTX Car3 Glut	1	1.000	0.000	0.036	0.000	0.800	0.667	0.000
054 STR Prox1 Lhx6 Gaba	1	0.000	0.000	0.000	0.000	0.000	0.000	0.000
177 SCig-an-PPT Foxb1 Glut	1	0.000	0.000	0.000	0.000	0.000	0.000	0.000
320 Astro-OLF NN	1	0.000	0.000	0.000	0.000	0.000	0.000	0.000
338 Lymphoid NN	1	0.000	0.000	0.015	0.000	0.000	0.000	0.000

Table S7 Per-cell-type F1 on the OB MERFISH dataset across seven competing methods. Support is the number of ST cells per cell type.

Cell type	Support	PRISM	DSCT	Cell2location	Tangram	RCTD	SpatialDWLS	Spatial-ID
041 OB-in Frmd7 Gaba	12034	0.967	0.936	0.672	0.741	0.978	0.864	0.000
328 OEC NN	3602	0.967	0.915	0.912	0.045	0.985	0.941	0.150
042 OB-out Frmd7 Gaba	2891	0.804	0.430	0.903	0.687	0.963	0.904	0.004
035 OB Eomes Ms4a15 Glut	1904	0.895	0.679	0.800	0.190	0.969	0.624	0.000
320 Astro-OLF NN	1349	0.706	0.655	0.626	0.222	0.882	0.686	0.080
044 OB Dopa-Gaba	1343	0.824	0.599	0.836	0.764	0.953	0.862	0.000
039 OB Meis2 Thsd7b Gaba	1255	0.849	0.518	0.759	0.413	0.951	0.765	0.000
333 Endo NN	1106	0.941	0.812	0.846	0.842	0.972	0.947	0.097
040 OB Trdn Gaba	801	0.729	0.641	0.595	0.663	0.883	0.573	0.000
045 OB-STR-CTX Inh IMN	695	0.828	0.795	0.687	0.499	0.889	0.625	0.020
330 VLMC NN	558	0.844	0.729	0.509	0.419	0.919	0.837	0.103
043 OB-mi Frmd7 Gaba	344	0.955	0.540	0.806	0.702	0.989	0.848	0.000
319 Astro-TE NN	334	0.240	0.140	0.239	0.169	0.078	0.121	0.006
327 Oligo NN	310	0.859	0.759	0.660	0.592	0.859	0.793	0.010
334 Microglia NN	159	0.914	0.725	0.570	0.579	0.871	0.791	0.000
329 ABC NN	158	0.703	0.611	0.563	0.240	0.870	0.789	0.000
326 OPC NN	153	0.902	0.631	0.685	0.693	0.872	0.777	0.005
331 Peri NN	136	0.765	0.466	0.478	0.839	0.898	0.912	0.002
332 SMC NN	41	0.663	0.462	0.249	0.737	0.851	0.843	0.000
065 IA Mgp Gaba	40	0.483	0.200	0.104	0.019	0.611	0.173	0.000
335 BAM NN	11	0.557	0.254	0.108	0.351	0.579	0.400	0.000
318 Astro-NT NN	7	0.026	0.028	0.029	0.000	0.333	0.006	0.000
066 NDB-SI-ant Prdm12 Gaba	5	0.027	0.011	0.014	0.000	0.429	0.000	0.000
337 DC NN	2	0.233	0.040	0.039	0.117	0.500	0.444	0.000
047 Sncg Gaba	1	0.600	0.398	0.026	0.900	1.000	0.222	0.000
049 Lamp5 Gaba	1	1.000	0.217	0.000	0.633	0.667	0.044	0.000
052 Pvalb Gaba	1	1.000	0.579	0.007	0.354	1.000	0.091	0.000
056 Sst Chodl Gaba	1	0.833	0.356	0.020	0.000	1.000	0.143	0.000
323 Ependymal NN	1	0.000	0.000	0.000	0.000	0.000	0.000	0.000
338 Lymphoid NN	1	0.477	0.215	0.016	0.314	0.000	0.000	0.000

Table S8 Per-metric label-free evaluation values across all eleven datasets used in Table 2 of the main text. RMSE and JSD: lower is better; Spearman ρ, Kendall τ, and CCC: higher is better.

Dataset	Method	RMSE	Spearman ρ	Kendall τ	CCC	JSD
MERFISH HIP (mouse)	PRISM	1.710	0.456	0.366	0.205	0.259
	Tangram	1.700	0.444	0.352	0.204	0.267
	RCTD	1.727	0.444	0.358	0.197	0.266
	DSCT	1.754	0.417	0.334	0.178	0.287
	Cell2location	1.742	0.364	0.291	0.154	0.325
	Spatial-ID	1.857	0.357	0.279	0.099	0.357
	SpatialDWLS	2.060	0.242	0.200	0.057	0.423
MERFISH OB	PRISM	1.794	0.428	0.343	0.169	0.283
	Tangram	1.851	0.382	0.306	0.138	0.308
	RCTD	1.782	0.418	0.338	0.166	0.289
	DSCT	1.839	0.345	0.277	0.122	0.334
	Cell2location	1.895	0.336	0.269	0.110	0.333
	Spatial-ID	1.882	0.366	0.288	0.110	0.349
	SpatialDWLS	1.823	0.324	0.262	0.116	0.353
MERFISH CTX_{mouse}	PRISM	2.285	0.431	0.342	0.157	0.271
	Tangram	2.275	0.429	0.340	0.156	0.274
	RCTD	2.349	0.414	0.328	0.144	0.277
	DSCT	2.296	0.418	0.331	0.152	0.277
	Cell2location	2.312	0.427	0.338	0.150	0.274
	Spatial-ID	2.358	0.391	0.307	0.129	0.302
	SpatialDWLS	2.516	0.346	0.270	0.106	0.314
MERFISH CTX_{human}	PRISM	0.663	0.217	0.176	0.207	0.460
	Tangram	0.666	0.208	0.169	0.206	0.463
	RCTD	0.663	0.177	0.144	0.167	0.494
	DSCT	0.665	0.218	0.176	0.202	0.462
	Cell2location	0.663	0.160	0.129	0.149	0.517
	Spatial-ID	0.689	0.161	0.131	0.138	0.491
	SpatialDWLS	0.671	0.172	0.139	0.160	0.495
Stereo-seq HIP	PRISM	0.218	0.072	0.061	0.143	0.623
	Tangram	0.218	0.073	0.061	0.141	0.626
	RCTD	0.217	0.061	0.052	0.112	0.644
	DSCT	0.215	0.070	0.058	0.124	0.633
	Cell2location	0.224	0.073	0.062	0.112	0.633
	Spatial-ID	0.231	0.069	0.058	0.065	0.652
	SpatialDWLS	0.220	0.054	0.047	0.081	0.655
STARmap HIP	PRISM	0.489	0.077	0.064	0.124	0.600
	Tangram	0.489	0.075	0.062	0.113	0.605
	RCTD	0.493	0.064	0.054	0.102	0.615
	DSCT	0.483	0.073	0.060	0.118	0.606
	Cell2location	0.496	0.071	0.060	0.087	0.618
	Spatial-ID	0.543	0.092	0.076	0.103	0.590
	SpatialDWLS	0.494	0.062	0.052	0.095	0.618
Slide-seqV2 HIP	PRISM	0.228	0.120	0.100	0.283	0.548
	Tangram	0.225	0.084	0.070	0.196	0.602
	RCTD	0.240	0.040	0.034	0.096	0.718
	DSCT	0.211	0.042	0.034	0.083	0.717
	Cell2location	0.224	0.063	0.054	0.100	0.670
	Spatial-ID	0.266	0.102	0.086	0.085	0.629
	SpatialDWLS	0.218	0.047	0.040	0.098	0.698
Stereo-seq CB	PRISM	0.519	0.116	0.100	0.190	0.575
	Tangram	0.485	0.116	0.097	0.189	0.586
	RCTD	0.531	0.112	0.096	0.178	0.583
	DSCT	0.491	0.098	0.083	0.154	0.598
	Cell2location	0.556	0.060	0.050	0.054	0.644
	Spatial-ID	0.517	0.102	0.086	0.092	0.606
	SpatialDWLS	0.530	0.095	0.081	0.141	0.607
MERFISH Liver	PRISM	1.899	0.390	0.315	0.322	0.304
	Tangram	2.013	0.202	0.164	0.152	0.386
	RCTD	2.130	0.317	0.254	0.247	0.341
	DSCT	1.943	0.370	0.298	0.299	0.316
	Cell2location	2.165	0.212	0.171	0.159	0.389
	Spatial-ID	2.066	0.267	0.214	0.151	0.352
	SpatialDWLS	1.966	0.358	0.288	0.292	0.319
Xenium BC (human)	PRISM	1.939	0.468	0.379	0.210	0.268
	Tangram	2.050	0.429	0.345	0.170	0.284
	RCTD	2.226	0.182	0.146	0.046	0.548
	DSCT	2.037	0.305	0.244	0.104	0.372
	Cell2location	1.876	0.419	0.338	0.186	0.315
	Spatial-ID	2.003	0.401	0.321	0.160	0.309
	SpatialDWLS	2.113	0.251	0.201	0.077	0.437
CosMx HCC (human)	PRISM	0.668	0.067	0.055	0.091	0.597
	Tangram	0.727	0.055	0.046	0.070	0.589
	RCTD	0.800	0.044	0.036	0.077	0.571
	DSCT	0.671	0.059	0.049	0.080	0.604
	Cell2location	0.806	0.043	0.035	0.058	0.572
	Spatial-ID	0.775	0.016	0.013	0.015	0.638
	SpatialDWLS	0.799	0.045	0.037	0.074	0.572

Table S9 Sensitivity to neighborhood size k on the HIP dataset.

k	SS ↑	KL ↓	Cosine ↑	Pearson ↑
0	0.0757	1224.2787	0.6000	0.5250
1	0.0760	1222.3257	0.5995	0.5243
5	0.0760	1220.8006	0.6002	0.5252
10	0.0759	1223.9435	0.6003	0.5252
**15**	0.0760	1221.5111	0.6004	0.5253
20	0.0759	1223.9284	0.6003	0.5254

Table S10 Sensitivity to neighborhood size k on the CTX_{mouse} dataset.

k	SS ↑	KL ↓	Cosine ↑	Pearson ↑
0	0.0853	774.8580	0.6373	0.4661
1	0.0853	775.9085	0.6376	0.4666
5	0.0855	774.0931	0.6377	0.4666
10	0.0855	774.6528	0.6382	0.4676
**15**	0.0856	773.7379	0.6383	0.4676
20	0.0855	774.1586	0.6381	0.4673

Table S11 Sensitivity to marker gene set size on the Stereo-seq dataset.

m	SS ↑	KL ↓	Cosine ↑	Pearson ↑
15	0.0788	216.7500	0.1664	0.1152
**30**	0.0950	211.0836	0.1907	0.1407
45	0.0909	204.0563	0.1876	0.1379

Table S12 Sensitivity to marker gene set size on the MERFISH dataset.

m	SS ↑	KL ↓	Cosine ↑	Pearson ↑
15	0.0749	1241.4445	0.5934	0.5153
**30**	0.0760	1221.5111	0.6004	0.5253
45	0.0728	1263.5374	0.5854	0.5042

Table S13 Sensitivity to marker gene set size on the Xenium dataset.

m	SS ↑	KL ↓	Cosine ↑	Pearson ↑
15	0.2117	330.6128	0.4643	0.3936
**30**	0.2180	323.0446	0.4742	0.4051
45	0.1979	335.4160	0.4610	0.3870

Table S14 Sensitivity to marker gene set size on the STARmap dataset.

m	SS ↑	KL ↓	Cosine ↑	Pearson ↑
15	0.1207	563.7288	0.2023	0.1537
30	0.1371	561.5200	0.2233	0.1746
**45**	0.1403	535.6905	0.2269	0.1803

Table S15 Sensitivity to marker gene set size on the Slide-seq dataset.

m	SS ↑	KL ↓	Cosine ↑	Pearson ↑
15	0.0814	141.1840	0.1647	0.1437
30	0.0932	142.4809	0.1809	0.1601
**45**	0.0929	133.9308	0.1902	0.1711

Table S16 Sensitivity to the number of pseudo-label aggregation rounds on the HIP dataset.

Rounds	Accuracy ↑	Std
1	0.877	0.003
3	0.906	0.002
5	0.905	0.001

Table S17 Sensitivity to marker gene prior weighting parameters γ and δ on the HIP dataset.

γ	δ	Accuracy ↑	Std
0	0	0.8734	0.0043
1	1	0.8985	0.0027
1	3	0.8981	0.0028
1	5	0.8995	0.0023
3	1	0.8984	0.0028
3	3	0.9013	0.0055
3	5	0.8981	0.0013
5	1	0.9055	0.0053
5	3	0.9050	0.0027
**5**	**5**	**0.9063**	0.0023
7	5	0.9015	0.0041
5	7	0.8957	0.0012

Table S18 Comparison of runtime and memory consumption on the **MERFISH HIP** dataset. **CPU/GPU** denote runtime in seconds (s). **RAM** and **VRAM** are measured in GB. **NA** indicates not applicable, i.e., CPU-only execution without GPU usage.

Method	CPU (s)	GPU (s)	RAM (GB)	VRAM (GB)
DSCT	88	25	30.8	2.0
Tangram	2117	141	27.1	15.0
Spatial-ID	10132	1081	87.5	3.5
Cell2location	103427	3234	50.2	5.3
RCTD	1501	NA	77.3	NA
SpatialDWLS	32357	NA	123.5	NA
**PRISM (Ours)**	2001	588	28.2	2.2
